# 3D printed scaffolds based on hyaluronic acid bioinks for tissue engineering: a review

**DOI:** 10.1186/s40824-023-00460-0

**Published:** 2023-12-24

**Authors:** Han Chen, Huaqian Xue, Huanxuan Zeng, Minghai Dai, Chengxuan Tang, Liangle Liu

**Affiliations:** 1 The Third Affiliated Hospital of Wenzhou Medical University, Wenzhou, 325200 China; 2 Ningxia Medical University, Ningxia, 750004 China; 3 Xijing Hospital of Air Force Military Medical University, Xi’an, 710032 China

**Keywords:** Hyaluronic acid, Bioink, 3D printing, Biomaterial, Tissue engineering

## Abstract

Hyaluronic acid (HA) is widely distributed in human connective tissue, and its unique biological and physicochemical properties and ability to facilitate biological structure repair make it a promising candidate for three-dimensional (3D) bioprinting in the field of tissue regeneration and biomedical engineering. Moreover, HA is an ideal raw material for bioinks in tissue engineering because of its histocompatibility, non-immunogenicity, biodegradability, anti-inflammatory properties, anti-angiogenic properties, and modifiability. Tissue engineering is a multidisciplinary field focusing on in vitro reconstructions of mammalian tissues, such as cartilage tissue engineering, neural tissue engineering, skin tissue engineering, and other areas that require further clinical applications. In this review, we first describe the modification methods, cross-linking methods, and bioprinting strategies for HA and its derivatives as bioinks and then critically discuss the strengths, shortcomings, and feasibility of each method. Subsequently, we reviewed the practical clinical applications and outcomes of HA bioink in 3D bioprinting. Finally, we describe the challenges and opportunities in the development of HA bioink to provide further research references and insights.

**Graphical Abstract:**

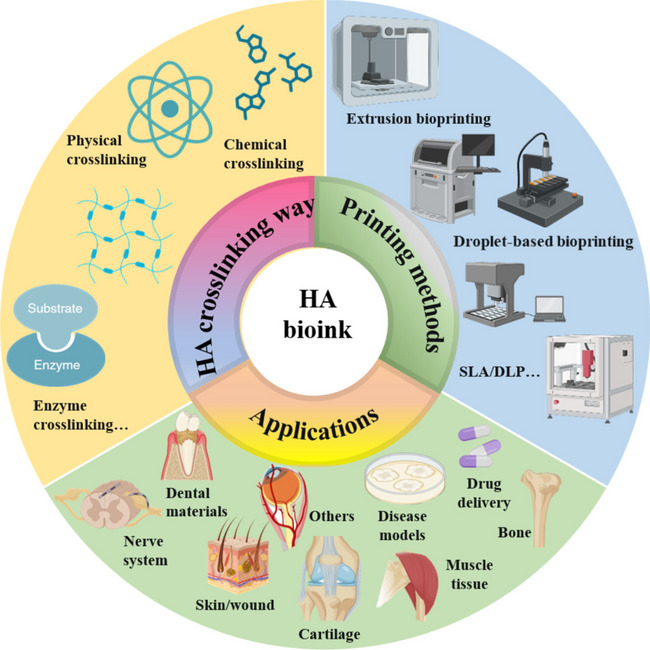

## Introduction

HA is a highly acidic polysaccharide that is widely distributed in the extracellular matrix (ECM) of human connective tissue. It has multiple physiological functions, such as maintaining cell structure and providing energy, and it also plays a significant role in embryonic development, stem cell differentiation, wound healing, and cancer progression [1]. This has led to its widespread use in biomedical research and clinical applications, especially in the field of three-dimensional (3D) bioprinting [2–4]. 3D bioprinting is defined as the deposition of biomaterial-based inks (bioinks) onto a solid or gel substrate or liquid reservoir in a layer-by-layer fashion using 3D printing technology. Bioinks must be extrudable through fine needles, and they should exhibit shear thinning and sufficient elasticity to maintain a 3D highly interconnected porous structure with appropriate mechanical characteristics. The stability of the final prints is also influenced by the gel time and cross-linking strategy. An ideal bioink should have optimized diffusivity, O_2_ and nutrients, waste permeability, and controlled biodegradability. More importantly, bioinks must have cytocompatibility. Typically, bioinks consist of a cell-loaded hydrogel. This emerging additive manufacturing technology uses artificial biomaterials, growth factors, living cells, bio-composite cellular materials, and other active ingredients to construct artificial tissues and organs with partial or complete biological functions, and it is also used to repair and replace tissues and organs in patients [5, 6].

Owing to the nature of its chemical structure, HA has hydroxyl and carboxyl-terminal functional groups that can be easily modified with different chemical modifications to enhance its physical and biological properties [7]. It can also be used in combination with other biomaterials, including natural and synthetic polymers. Synthetic polymer bioinks produce stiffer bio-prostheses but are less biocompatible than natural polymers [8]. Moreover, the chemical composition of HA can be easily modified via the use of various cross-linking chemical mechanisms that have been developed to improve bioactivity. This allows HA bioinks to support gelling capabilities and improves their cytocompatibility, making them suitable bioink components for bioprinting.

Several studies have used cells (e.g., mesenchymal stem cells (MSCs), primary cells, and neural precursor cells) or signaling molecules (e.g., carbon nanotubes, growth factors, and hydroxybenzoids) as additive components of HA hydrogel bioinks [9]. These approaches have been successful in promoting the regeneration of smaller tissues; however, the regeneration of large tissues remains a challenge due to issues associated with supplying blood and nutrients to newly developed tissues [10]. Thus, further investigation is required. HA bioinks are widely used in tissue engineering and regenerative medicine. This review focuses on cartilage tissue engineering, neural tissue engineering, wounds, neural tissue engineering, and so on, in which researchers have made a lot of cutting-edge innovations and discoveries in the development of inks, scaffolds, and mechanisms to shorten the distance between scientific research and the clinic.

In this review, we report the definition, synthesis, and degradation of HA. It is important to understand that HA synthesis and degradation are critical for the development of bioinks and that the stability and degradation of printed material are crucial for practical applications [7]. This review provides an overview of the unique physicochemical and biological properties of HA that are critical for the development of HA-based tissue engineering scaffolds. We then provide a detailed description of the sources of commercial HA, including animal, microbial, and synthetic sources, compare their advantages and disadvantages, and discuss how the easy availability, relatively low cost, and high purity of HA raw materials are useful for the development of HA bioinks [11]. Next, we evaluate different methods and strategies used to modify and crosslink HA and discuss the advantages of different HA-based bioinks. Subsequently, we summarize the 3D printing strategies used for HA bioinks and compare their advantages and disadvantages [7]. In particular, this review highlights the description of the exclusive properties of HA polymers used as bioinks for 3D bioprinting and their successful applications in the field of regenerative medicine, and it also discusses the relevant mechanisms at play. Finally, we outline the future prospects and current challenges of using HA-based biomaterials for 3D bioprinting in tissue engineering and regenerative medicine. We intend for this work to provide a reference for researchers.

## Definition and origin of HA

### Definition

HA is a highly polymerized macromolecular mucopolysaccharide that includes repeating disaccharide units composed of d-glucuronic acid (d-GlcA) and *N*-acetylglucosamine (GlcNAc) with alternating β-1,3 and β-1,4 glycosidic bonds [12]. HA was first isolated by K. Meyer, a biochemist at Columbia University, and his assistant J. Palmer in 1934. They isolated this polymer from the bovine vitreous and named it “hyaluronic acid,” which was derived from the words “hyaloid” (after the hyaloid of the vitreous) and “uronic acid” (due to its high uronic acid content). Šoltés et al. [13] discovered that HA is also contained within the pods formed by some bacteria (e.g., streptococci) and provides adhesive and protective properties and promotes molecular mimicry to evade the host immune system during infection [13, 14].

### Sources and production

During the 1930s, HA was successively isolated from various tissues and organisms, such as the vitreous humor, corpuscles, umbilical cords, and streptococci [15]. Current commercial HA is often extracted through the following methods: animal issue extraction, microbial fertilization, and enzymatic synthesis. Several animal tissues are used for HA extraction, including bovine vitreous humor and synovial fluid, umbilical cords, and cockscombs. The highest reported HA concentration of up to 7500 μg HA/g [15] is found in the cockscomb. However, HA extraction from animal tissue is challenging because HA remains bound to cellular proteins, such as HAS, and these contaminants can induce abnormal immune responses [16]. Finally, HA extraction from animal tissues is costly, slow, and labor-intensive and presents an extremely low extraction rate and complex separation and purification processes [17]. Costly purification protocols are required to reduce the risk of toxin contamination, which may lead to cross-contamination [18]. Research on HA production via microbial fermentation began in the 1980s and has rapidly developed in recent decades [19]. Producing HA from microorganisms offers the opportunity to create more economical, direct, raw-material-free, and environmentally friendly methods. HA is mainly produced by Lancefield streptococci A and C, especially *Streptococcus zooepidemicus* and *Streptococcus equip*. Microbial production by fermentation uses glucose as a carbon source, and fermentation proceeds in a fermentation broth. Batch culturing is the most common fermentation method for HA production [20]. The downstream treatments can be combined with synthetic biology approaches, such as cell flocculation in the late stages of fermentation, which can reduce the cost of HA production [21]. The use of enzymes for in vitro synthesis is another promising technique for the production of high-purity HA with adjustable molecular weight, contaminant-free synthesis, and batch-to-batch invariability. However, enzymatic synthesis has not yet reached the commercial scale [22]. The in vitro preparation of HA has also become possible with the development of bioprocessing and chemical enzymatic synthesis technologies. DeAngelis et al. [23] first reported the cloning of HAS and related gene clusters from *S. faecalis* in 1993, thereby achieving the heterologous synthesis of HA. HAS catalyzes d-glucuronide (UDP-GlcUA) and *N*-acetylglucosamine (UDP-GlcNAc) in vivo to produce HA. The synthesis of HA by artificial enzymes has unique advantages, such as good product uniformity, mild reaction conditions, and environmental safety [24]. However, owing to the time-consuming and laborious nature of this manual synthetic process and the unsatisfactory control over end-product formation, it is not considered an ideal method. Moreover, the synthesized substances are expensive and difficult to apply for industrial processes; therefore, these methods are only suitable for the production of high-molecular-weight, high-purity HA with special applications [25].

### Synthesis and degradation

The biosynthesis of HA begins with monosaccharide enzymes, wherein the monosaccharide substrates UDP-GlcUA and UDP-GlcNAc are formed via the tricarboxylic acid cycle and pentose phosphate pathways. The two substrates are then linked to the structural unit of HA by the key enzyme hyaluronidase (HAS), which releases progressively longer sugar chains into the cell [26]. Some precursors in HA biosynthesis are shared with other metabolic pathways, such as the glycolytic, pentose phosphate, and lactate metabolic pathways [18, 27]. The in vivo degradation of HA can be viewed as a depolymerization process, which involves the breakage of glycosidic bonds, and is mainly accomplished by two mechanisms: enzymatic and free radical degradation. The in vivo enzymatic hydrolysis of HA is mainly accomplished by the action of the HAS family, which has six members: HYAL-P1, PH-20, HYAL-1, HYAL-2, HYAL-3, and HYAL-4. Among these, the most active enzymes are HYAL-1 and HYAL-2 [28, 29]. The degradative metabolism of HA occurs in situ (e.g., in the ECM) and in cells and lymph nodes. The in situ degradation of long-chain HA by enzymes and free radicals produces smaller HA oligosaccharides, which are then further degraded and metabolized in cells and lymph nodes and eventually enter the circulatory system, wherein they are eliminated by the liver and kidneys. This indicates that HA has good degradability and biocompatibility [30, 31]. Additionally, HA undergoes isotropic degradation, which increases the distance between molecules, and thereby enabling more water to be absorbed. This property allows HA to retain its stability over long periods [32, 33].

### Physical properties

The molecular structure of HA has a rigid single- or double-helical spatial configuration owing to hydrogen bonding between groups on the axis of the straight chain. In a dry state, HA is an odorless white amorphous powder with strong hygroscopicity and solubility in water but insolubility in organic solvents. The conformation of HA varies with the properties of the external environment (e.g., concentration and ionic strength) [34]. In aqueous solution, HA has a rigid, random nematic structure. HA chains become entangled at higher concentrations, thus forming a continuous 3D network with unique rheological properties. As the HA concentration increases, the inter- and intramolecular interactions also increase, thereby resulting in the formation of a network structure. HA is naturally negatively charged because of its carboxylic acid groups. Its negative charge enables it to bind with large amounts of water to form highly viscous anionic gel-like polymers [35]. Owing to its negative charge, HA molecules are in a relaxed state and occupy a large amount of space due to electrostatic repulsion. In addition, the negative charge allows HA to bind cations in large quantities, and these bound cations can also bind large numbers of water molecules [36].

### Rheological properties

HA aqueous solutions are non-Newtonian fluids with unique rheological properties, and they have a reticulated structure that makes them simultaneously viscous and elastic, that is, uniquely viscoelastic [37]. The molecular weight of HA ranges from 10^5^–10^7^ Dalton (Da) depending on the source. HA with a molecular weight of ≥ 10^6^ Da forms a viscous aqueous solution that exhibits good hydrating activity on cultured human skin cells. Additionally, it has unique biocompatibility and viscoelasticity that are useful for ophthalmology, cosmetics, and orthopedic wound healing [38]. HA with a molecular weight of 10^4^–10^6^ Da is beneficial for the development of crosslinking products. Finally, HA with a molecular weight of ≤ 10^4^ Da can provide deep hydration, long-lasting moisturization, increased skin elasticity, and enhanced dermal water storage [39]. A current challenge for HA applications in the field of bioinks is the balance between the rheological properties required for printing and the physicochemical properties required for printing substrates, such as the complex relationship among rheological parameters, cell viability, and printability [40]. In addition, the viscoelastic performance of bioinks has been shown to be vital for maintaining cell viability during the printing process. For extrusion-based 3D printing, one of the most essential properties of bioinks is the shear-thinning behavior, in which material flows under high shear stress but maintains a significantly higher viscosity under low shear conditions [41, 42]. To obtain printable bioinks, several researchers have focused on developing formulations based on the original HA, HA derivatives, and various combinations of synthetic and natural polymers. HA with molecular weights between 120 and 2,500 kDa and HA polymers with concentrations between 0.1% and 4% w/v have been used for 3D printing to generate constructs with large specified mechanical properties and biodegradation rates [43, 44].

### Biological activity

HA is a non-sulfated glycosaminoglycan that is distributed in the ECM of epithelial, connective, and neural tissues. HA is inherently biocompatible and nonimmunogenic and plays an important role in various biological processes, including wound healing, vascularization, and the activation of various signaling pathways [6]. The ECM provides structural support for the normal physiological activity of tissue cells and plays an indispensable role in immune regulation in both homeostatic and pathological states owing to its abundant content of proteins and immune-active molecules. For example, the HA-mediated motility receptor RHAMM, an HA-binding protein located in the cytoskeleton and centrosome, has diverse functions, and its increased expression plays a major role in tumorigenesis by inducing genomic instability and cancer progression [45]. CD44 is a HA receptor and cell adhesion molecule. Both CD44 and RHAMM enable cell adhesion and proliferation on HA. HA can preserve tissue architecture and also promote angiogenesis by activating the endothelial cell (EC) surface receptor ICAM to impact cell behaviors, such as growth and migration [20, 21]. In addition, HA can be degraded through the receptor-mediated uptake of collagen into lysosomes. For example, HA interacts with macrophages expressing LYVE-1 to initiate MMP9-mediated collagen degradation and regulates the composition of the arterial ECM to maintain arterial tone and diameter [22] The diffusable nature of HA-based hydrogels makes them attractive candidates for the simulation of soft tissue microenvironments and as reservoirs for growth factor delivery and water-soluble cytokines.

## Modification of HA

**Fig. 1:**
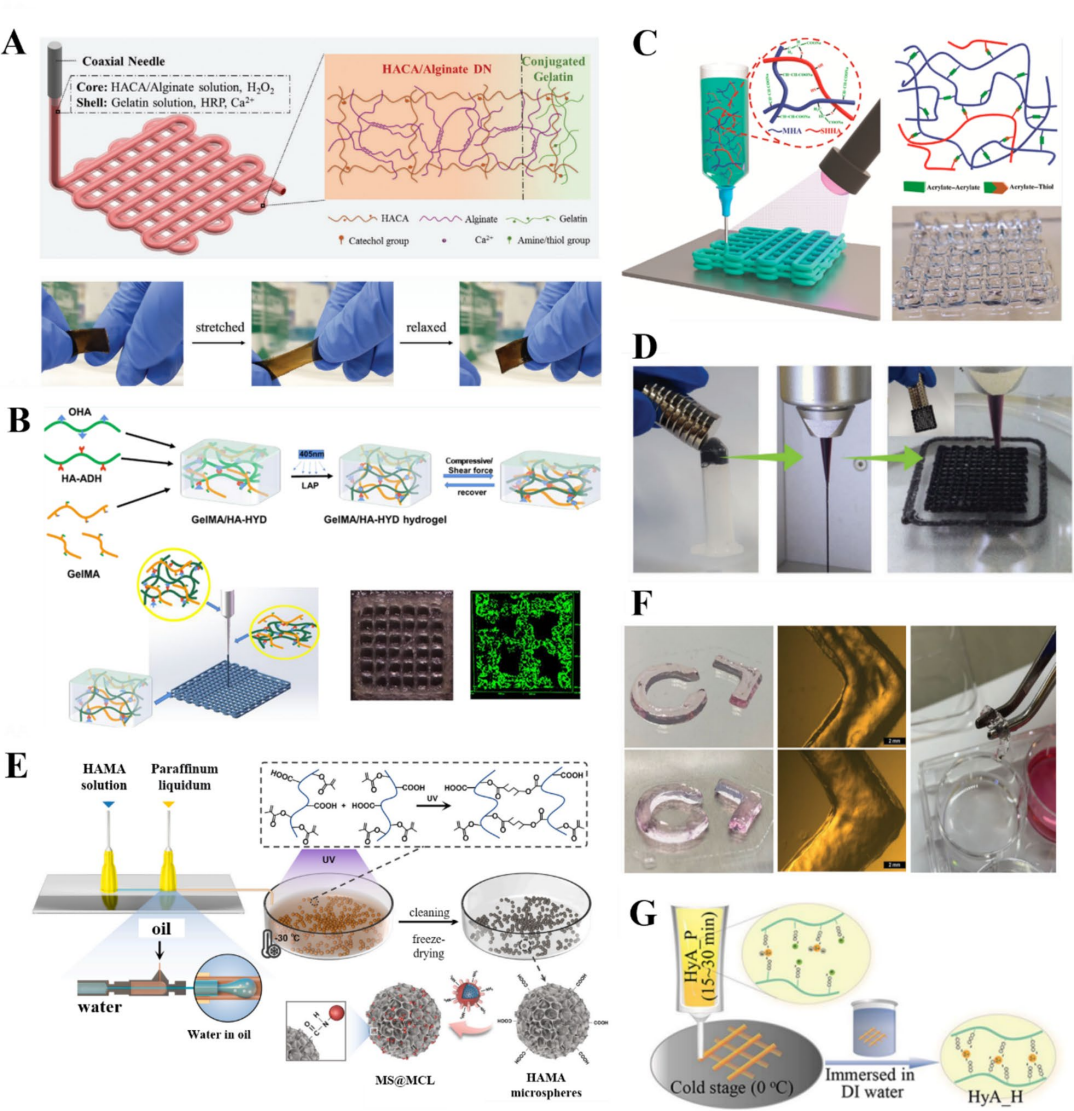
Functions of HA-based hydrogels. **A** Catechol-modified HA/alginate double network hydrogels with high fracture toughness and elasticity for 3D coaxial printing [51]. Copyright 2020, Wiley–VCH GmbH. License Number: 5618121300533. **B** Gelatin GelMA/HA-dynamic hydrazone (HYD) hydrogel bioinks that produce mechanically strong printed structures [52]. Based on Creative Commons Attribution-Non-Commercial International Public License (CC BY-NC). Copyright 2022, the Authors. Published by American Chemical Society. **C** Sodium-maleated HA (MHA)/sodium-HASA hydrogel precursors with fast gelation kinetics. Reprinted (adapted) with permission from [53] .Copyright 2022, American Chemical Society. **D** Magnetically responsive Nanoclay-Incorporated Double-Network (NIDN) hydrogels for 3D printing [54]. Copyright 2021, Wiley–VCH GmbH. License Number: 5618161075332. **E** Microfluidic preparation of injectable HAMA microspheres [55]. Copyright 2021, Elsevier B.V. All rights reserved. License Number:5618161075332. **F** Thiol-functionalized hyaluronic acid dynamic hydrogel with Au ions for better printing accuracy and applicability [56]. Copyright 2022, Wiley–VCH GmbH. License Number: 5618170397422. **G** Adjustable crosslinked HA gel for 3D printing. Reprinted (adapted) with permission from [57]. Copyright 2021, American Chemical Society

The wide range of applications and large market demand of HA have led to its successful commercialization; however, its short half-life has hindered its clinical application. The susceptibility of HA to degradation limits the application of natural HA-based hydrogels in 3D bioprinting applications because it can change the mechanical properties of the hydrogel, such as the stiffness. Fortunately, the structural modification of HA facilitates the development of HA-based hydrogels with tunable mechanical and biological properties [23]. More importantly, the abundant hydroxyl, carboxyl, and amino groups on the HA side chain facilitate the preparation of functional HA-based precursors, which are beneficial for the development and application of more bioink materials. More importantly, the abundant hydroxyl, carboxyl, and amino groups on the HA side chain facilitate the preparation of functional HA-based precursors** (**Fig. 1A**)**, which are beneficial for the development and application of more bioink materials [24]. Several HA modification strategies have been reported for different applications. The chemical modification of HA enables the fabrication of HA-based hydrogel scaffolds with different shapes, morphologies, and biophysical and biochemical properties** (**Fig. 1B**)**. Such modifications have been exploited in the development of HA-based bioinks that undergo crosslinking and stabilization after extrusion [25]. HA is usually chemically modified by the addition of functional groups, such as glucuronide and primary and secondary hydroxyl (*N*-acetylamino) groups. The reduced end of HA can also be modified, although this method is less frequently used. The resultant HA derivatives have high molecular weight but still retain the original physicochemical properties of HA. They also have low toxicity [46], good biocompatibility [47], and resistance to HAS degradation [48]. These characteristics allow HA to remain in the body for a long time and stabilize its unique physicochemical properties [49]. Moreover, the hydrogels can be tailored to specific clinical applications and 3D bioprinting methods [50]. Synthetic hydrogels have good gel kinetics and mechanical tunability, which facilitates bioprinting; however, their viscoelastic properties and mechanical properties observed after printing can be challenging to adjust. Nonetheless, the gel kinetics and mechanical tunability characteristics make bioink materials more personalizable for different applications **(**Table 1**)**.

**Table 1:** HA-based bioinks for different tissue-engineering applications

Material	Cells/Active substance	Crosslinking	Applications	In vitro /In vivo	Bioprinting technology	Ref
GelMA/HAMA	Chondrocytes	/	Cartilage	both	DLP printing	[58]
HA-TYR	Chondrocytes	Enzyme crosslinking	Cartilage	in vivo	Extrusion-based bioprinting	[59]
HA	rhbmp-2, Kartogenin, TCP	Chemical crosslinking	Cartilage	in vivo	Extrusion-based bioprinting	[60]
Gel/HA/PVA	DX	Physical crosslinking	Bone	in vivo	Extrusion-based bioprinting	[61]
Gel/HA/HyA	Mscs	Chemical crosslinking	Bone	in vitro	Extrusion-based bioprinting	[62]
HA/PLL	c(RGDfC)/β-TCP	Physical crosslinking	Bone	in vitro	Extrusion-based bioprinting	[63]
PCL-PU/HAMA	C2C12	Chemical crosslinking	Skeleton muscle	in vitro	Extrusion-based bioprinting	[64]
HACA/Alg	C2C12	Combining multiple crosslinking	Skeleton muscle	in vitro	Extrusion-based bioprinting	[51]
HAVS/HASH/HBC	Nscs	Combining multiple crosslinking	Neural tissue	both	Extrusion-based bioprinting	[65]
PVA/HA-PBA	Neural precursor cells	Chemical crosslinking	Neural tissue	in vitro	Extrusion-based bioprinting	[66]
GelMA/HAMA	Human dermal fibroblasts	Chemical crosslinking	Skin	in vitro	LAB bioprinting	[67]
HA/PCL/45S5 Bioglass	hDPSCS	/	Dental materials	in vitro	Extrusion-based bioprinting	[68]
RSFMA/HAMA	hDPSCS	Chemical crosslinking	Dental materials	in vitro	Extrusion-based bioprinting	[69]
Collagen/HA	Patient-derived cancer cells	Physical crosslinking	Disease models	in vitro	Extrusion-based bioprinting	[70]
HAMA	Islet cells/Pancreatic ECM	Combining multiple crosslinking	Disease models	Both	Extrusion-based bioprinting	[71]
Chitosan/HA	Vancomycin	Chemical crosslinking	Drug delivery	in vitro	Extrusion-based bioprinting	[72]
HA	HUVECs/Fibrinogen	/	Vascularized tissue	in vitro	Extrusion-based bioprinting	[73]
Gel/HA/Elastin	Ocular epithelial cells	Chemical crosslinking	Corneal tissue	Both	Extrusion-based bioprinting	[74]

### HA esterification

The esterification of HA is a two-step process. First, the hydroxyl group in the HA structure is esterified with an acid or anhydride to obtain esterified HA. Second, the carboxyl group in the HA structure is reacted with alcohols, phenols, epoxides, or halogenated hydrocarbons to form esterified HA derivatives. The carboxyl group of HA, which is responsible for the negative charge, is the most important site for modification [75]. Pure HA lacks printability and therefore cannot be used as a bioink for 3D printing. Hyaluronic acid methacrylate (HAMA) is a biocompatible methacrylated product of HA. Schuurmans et al. [76] obtained esterified HAMA by reacting HA with excess methacrylic anhydride at pH 8–9 and 4 °C. HAMA can be further crosslinked with polyamino acids under ultraviolet (UV) irradiation to form a hydrogel with a network structure that provides sufficient mechanical properties to enable it to be used as a bioink for biomedical applications. HAMA is a new type of 3D printing hydrogel ink with fast photosensitive response, fast gelation speed, and stable hydrogel performance. HAMA hydrogels have no cell adhesion sites but can be modified by a simple photopolymerization reaction with arginine–glycine–aspartate peptides to impart cell adhesion, extension, and proliferation abilities. Koivusalo et al. [77] grafted dopamine moieties onto crosslinked HA hydrogels to impart tissue-bonding properties. The resultant hydrogel exhibited good tissue adhesion upon implantation and could bind to adhesive protein peptides, which facilitated cell adhesion. Hossain [78] fabricated 3D cell-content hydrogel scaffolds by combining it with gelatin methacryloyl (GelMA) to overcome the lack of cellular adhesion of HAMA. Compared to HAMA alone, the hybrid bioink showed a 55% increase in stiffness, achieved cell adhesion, and maintained high cell viability. This research provides a firm foundation for the development of a stable hybrid bioink with HAMA and GelMA that can be used for stereolithography (SLA) 3D bioprinting.

### HA amidation

The carboxyl group of HA can also be modified by amidation to form HA-based amide compounds. This approach allows for the introduction of additional chemical groups to produce different HA derivatives. Yang et al. [79] modified HA via amidation with ethyl cysteine to produce cystamine-modified HA. Benzaldehyde-functionalized polydopamine-polypyrrole nanocomposites have also been prepared, resulting in hydrogels with good injectability, excellent tissue adhesion, self-healing properties, in vivo hemostatic ability, good antioxidant properties, and electrical conductivity. A HA-tyramine (HA-TYR) derivative was generated by the amidation of HA with Tyr residues using DMTMM, a coupling agent. The introduction of peptide amphiphiles promotes cell adhesion, angiogenesis, and osteogenesis [80].

### HA ring-opening modification

The main chain of HA undergoes a ring-opening reaction in the presence of oxidizing agents to produce aldehyde-capped HA derivatives with new modification sites. For example, when sodium periodate, a strong oxidizing agent, is added to an aqueous solution of HA, a dialdehyde-capped ring-opened HA derivative is produced. HA ring-opening improves the flexibility of the polymer backbone and the viscoelasticity of HA-based gels, which improves their mechanical strength and degradation resistance. Chen et al. [81] evaluated the chemical decomposition of HA with sodium periodate to introduce an aldehyde group and then performed acylation to introduce a methacrylic group, which resulted in an aldehyde-based HAMA with photocuring ability. The resultant molecule was rich in aldehyde groups and could chemically bond to cartilage tissue with good adhesion, and the inherent photocuring ability of HAMA allowed for rapid in situ gelation. HA is very sensitive to enzymatic degradation in the presence of HAS, which reduces its molecular weight. This has been used to produce aldehyde-capped ring-opened HA:HA oligomers containing double bonds via HAS degradation; subsequently, ozone decomposition is used to form an ozone oxide, which is finally reduced by a reducing agent to obtain aldehyde-capped ring-opened HA [82]. The terminal aldehyde group can react with the amino groups of vitamins and proteins via Schiff base reactions. The resulting products are promising for use in tissue repair.

### HA hydrazine group modification

Hydrazine modification is a type of chemical modification that can enhance the mechanical strength of HA hydrogels. HA has been modified with hydrazine groups, such as by reactions with adipic dihydrazide in the presence of 1-ethyl-3-(dimethylaminopropyl) carbodiimide hydrochloride, and then used to produce products, such as hyaluronic acid norbornene (HANB), which is a hydrophilic photocurable bioink [83, 84]. HANB hydrogels have good cytocompatibility, excellent cell proliferation, and high bio-orthogonality. The introduction of a near-infrared-soluble crosslinker consisting of a coumarin derivative and polyethylene glycol spacer allows for the preparation of photodegradable hydrogels with porous structures [85]. Ren et al. [86] constructed hydrogel adhesives by OPA/N-nucleophilic condensation reaction with hydrazide-modified HA and four-armed polyethylene glycol (4aPEG-OPA) at the end group of OPA as the building blocks, which could achieve a strong adhesion of the hydrogel to tissues through the formation of stable phthalimide bonds. Due to its strong tissue adhesion and coagulation effects, 7% (w/v) HA-PEG hydrogel was evaluated as a potential sealant to achieve hemostasis in a rat hepatic hemorrhage model as well as a rabbit femoral vein and artery hemorrhage model.

### HA sulfonation

In HA sulfonation, the hydrogen atom of HA is replaced with the sulfonic acid group (–SO_3_H) of sulfuric acid. Sulfonation is an effective method of chemically modifying HA** (**Fig. 1C**)** that not only improves the in vivo stability of HA, but also greatly enhances its biological functions, such as anti-inflammation and the promotion of physiological cell proliferation [87]. Highly sulfonated HA has a strong anti-inflammatory response and is able to regulate and maintain contractile smooth muscle cells; therefore, it has good potential for vascular tissue engineering applications. Wu et al. [88] constructed a “simulated skin” flexible hydrogel from polyaniline and sulfonated HA. The hydrogel exhibited similar electrical conductivity to that of skin and produced good electrical stimulation. Moreover, it was effective for promoting the healing of chronically infected wounds.

### HA vulcanization

HA vulcanization is the chemical process of sulfur crosslinking and involves the attachment of one or more sulfur atoms to the HA polymer chain to form a bridge-like structure [89]. HA vulcanization produces an elastomer with markedly different properties to those of the original material and offers better hydrogel stability and mechanical properties. Xia et al. [90] obtained a thiolated HA (HASA) derivative by vulcanization, added it to an acrylated nanogel system, and then performed rapid hydrogel formation. This new biodegradable gel had good biological selectivity and fast degradation. Li[91] designed hybrid hydrogels consisting of HASH and collagen type I of different molecular weights to investigate the chondrogenic differentiation of rabbit bone marrow mesenchymal stem cells. Increasing HASH molecular weight reduces the mechanical properties of hybridized hydrogels but improves viscosity and resistance to degradation, which can modulate cell spreading and morphological changes that affect cartilage differentiation. Wirostko et al. [92] prepared carboxymethylated HA and then introduced crosslinked thiol residues to produce sulfide-modified carboxymethylated HA hydrogels. The rheological properties, pore size, molecular diffusion rate, and chemical properties of the hydrogel could be modulated by varying the degree of crosslinking. This sulfide-modified HA derivative exhibited good mucosal adhesion and a low biodegradation rate.

## Crosslinking of HA

HA and its derivatives are beneficial for their biological relevance, cytocompatibility, shear-thinning properties, and tunability through chemical modification [93]. However, HA-based hydrogels often degrade rapidly and have poor mechanical properties. One approach for overcoming these limitations is by chemical modification. Another attractive strategy is HA crosslinking, which has been extensively investigated for the development of HA-based hydrogels. Crosslinked HA hydrogels have a dense mesh structure, wherein the HA macromolecules are highly aggregated and locally folded. This makes them less susceptible to degradation, thereby prolonging their half-life [94, 95]. In addition, for 3D bioprinting, crosslinked HA hydrogels can be loaded with bioactive agents to improve the printing precision and mechanical strength of the printed scaffolds. Both physical and chemical crosslinking methods have been explored in the design of HA-based biomaterials. The crosslinking concentration or density determines the physical properties of the hydrogel, including its diffusivity, elasticity, mesh size, magnetic responsiveness** (**Fig. 1D**)**, and water content [96]. The degree of crosslinking affects the degradation rate of the hydrogel. Therefore, it is essential to precisely control the crosslinking density of the hydrogel. Moreover, HA bioinks must exhibit shear-thinning behavior to be squeezed through the thin nozzle tip. In addition, they must have sufficient elasticity to retain a 3D structure with highly interconnected pores and appropriate mechanical characteristics. The final stability is also influenced by the gelation time, which typically depends on the crosslinking strategy [97]. Hydrogel crosslinking is often controlled by click chemistry, which minimizes side reactions and toxicity [98]. This section discusses several notable developments in HA crosslinking.

### Physical crosslinking

Physical crosslinking interactions include ionic and electrostatic interactions, chain entanglement, hydrogen bonding, van der Waals interactions, and hydrophobic self-assembly. Physically crosslinked hydrogels can be easily prepared without the use of potentially toxic chemical crosslinkers or initiators [99]. In addition, they offer several benefits for use as bioinks, including dynamic crosslink exchange, shear-thinning behavior, and excellent shear recovery. However, they often have insufficient mechanical strength owing to the low strength of the physical crosslinking interactions and their degradation rate is difficult to control [100]. The physical crosslinking of HA leads to rapid gelation; however, the gel can disassemble upon swelling and in the presence of competitive ions, such as negatively charged biopolymers (e.g., heparan sulfate) in biological environments. Furthermore, the hydrogels produced by these methods cannot be easily fine-tuned because their properties are heavily dependent on the inherent properties of the polymer itself [101]. Ester benzene-substituted HA is a negatively charged polyanionic electrolyte that adopts a self-assembled physically crosslinked network. It can self-assemble with positively charged poly-lysine layers to form a multilayer membrane, which can increase the degree of crosslinking, enhance the rigidity of the hydrogel, and improve its adhesion to cells [102].

### Chemical crosslinking

Chemical crosslinking offers more options for preparing crosslinked networks than physical crosslinking. For instance, it can be achieved by introducing chemical crosslinking agents. It can also be cross-linked by chemical reaction like performing Schiff base reactions, athiol–vinyl sulfone and thiol–maleimide Michael addition reactions, zide–alkyne and azide–alkyne cycloaddition reactions, and hydrazone and oxime formation reactions [98]. Enzymatic crosslinking is also one of the options available. Moreover, these approaches promote better matrix stability than physical crosslinking because of the higher flexibility and spatiotemporal control of the hydrogel network [103]. However, complex modification techniques or chemical crosslinking agents are usually required, which may affect the biological functionality or cytotoxicity of the hydrogel. Crosslinking of HA hydrogels with chemical crosslinking agents may cause cytotoxicity and immune responses in the host [104].

#### Chemical crosslinking agents

Injectable-grade HA produced by microbial fermentation can be crosslinked using a chemical crosslinking agent, such as 1,4-butanediol diglycerol ether, to form a stable 3D network. The crosslinks are irreversible bonds and thus are considered “permanent.” These biological materials are used to develop cosmetic products, such as volumetric dermal fillers, through a series of processes, including purification and sterilization, to obtain granulated crosslinked HA gels [105, 106]. Wang et al. [69] synthesized HAMA by grafting methacrylic anhydride onto the HA backbone. HAMA was then combined with a vinyl-modified filament protein (RSFMA) and a suitable amount of photo-initiator (I2959), which enabled photo-initiator-driven crosslinking to form a hydrogel scaffold under UV light irradiation. This system overcomes the shortcomings of conventional crosslinking methods and provides a hydrogel system with spatiotemporal control of the hydrogel network. Injectable HAMA microspheres can also be grafted with nanozymes by covalent bonding to the microspheres** (**Fig. 1E**)**. However, HA lacks the gelation ability necessary for maintaining its 3D structure after 3D printing. Therefore, a number of studies have focused on combining HA with natural gelling agents to improve the gelation ability without using toxic materials. For example, Antich et al. [107] designed a new HA-based bioink with alginate (Alg; a biomaterial with biocompatibility, good mechanical properties, and fast gelation kinetics) for the 3D bioprinting of hybrid structures for cartilage regeneration. When dopamine-modified HA is combined with a dynamic crosslinking agent, it forms dynamic borate ester bonds between microgels, resulting in a dynamic crosslinked microgel assembly. The addition of dynamic crosslinkers improves the mechanical strength of the bioink while retaining its shear-thinning properties [108].

#### Chemical reaction crosslinking

Dynamic covalent coupling is widely used in the development of HA-based microgels because it offers a gentle, efficient, and biocompatible strategy without releasing toxic byproducts. Moreover, the physical and mechanical properties of the hydrogels can be adjusted without impairing their biocompatibility [109, 110]. HA-based hydrogels with dynamic covalent coupling have been used in various biomedical applications. Similar to azide–alkyne and azide–alkyne cycloaddition reactions, strain-promoted [3 + 2] cycloaddition is used to form biocompatible crosslinked hydrogels from azide- or cyclooctyl-modified HA. HANB supports thiolene click crosslinking via spatially controlled photo-initiation. Maleimide-modified HA can be directly crosslinked with dithiols via Michael addition reactions, such as in the case of cysteine-containing peptides. Aldehyde-functionalized HA and the non-ECM component N, O-carboxymethyl chitosan can be crosslinked by Schiff base reactions [111, 112]. Elective Diels–Alder reactions have also been used to crosslink furan- and maleimide-functionalized HA. Hydrazone and oxime formation reactions have also been employed, such as hydrazine crosslinking of HA hydrogels, in which carbodihydrazide is used as the hydrazine group and hyaluronic aldehyde is used as a substrate for recombinant human bone morphogenetic protein-2 [113]. Ion replacement reactions include HA dynamic hydrogels technology based on metal (e.g., Au) sulfate/disulfide exchange reactions [56]. Notably, the interaction between sulfhydryl groups and Au ions minimizes the oxidation of sulfhydryl groups to disulfides at physiological pH levels **(**Fig. 1F**)**. These methods have been used to design HA-based polymers with ideal rheological properties for 3D printing, and they do not affect the nucleophilic nature of the permanent exchange of sulfhydryl groups with disulfides. A supramolecular hydrogel based on HA and recombinant peptides was prepared using metal–ligand bonding to enhance the capture and release performance of bioactive constituents [114].

#### Enzyme crosslinking

Flegeau et al. [59] prepared HA-TYR hydrogels with enzymatic crosslinking by adding horseradish peroxidase and hydrogen peroxide. The density and size of the microgel were adjustable, thus enabling control over the sample porosity, yield stress, shear thinning, and shear recovery and making it suitable for extrusion-based 3D printing applications. However, reducing the microgel density increased the porosity and reduced the printability and rheological properties. Li et al. [115] utilized bacterial transpeptidase sortase A (SA) to prepare HAMA hydrogels, which show rapid gel kinetics at high SA crosslinking concentrations and can be used as injectable hydrogels for tissue repair or extrusion-based bio-3D printing.

### Combining multiple crosslinking methods

To achieve high-resolution hydrogel scaffolds with superior structural flexibility, bioinks are often prepared by combining multiple crosslinking methods to enhance their biological properties and physical strength, thereby increasing the usefulness of the bioinks for different areas of tissue engineering. Wan et al. [53] prepared HA-based hydrogels by co-modifying sodium-maleated and sodium-thiolated HA by thiol–acrylate Michael addition pre-crosslinking. Subsequently, thiol–acrylate and acrylate–acrylate photopolymerization were used to covalently crosslink the hydrogel precursors. This method provided rapid gelation kinetics and increased compressive strength. Janarthanan et al. [42] developed hydrogel bioinks by crosslinking Alg and HA through various mechanisms, including hydrazine interactions, acyl–hydrazone reactions, and calcium-ion crosslinking agents. These Alg-HA hydrogels exhibited good shear-thinning ability, dynamic tunable mechanical properties, and excellent biocompatibility. Chen et al. [116] applied a kinetic interlocking multiunit strategy to an HA network by introducing different supramolecular motifs in an organized and alternating manner, and they successfully increased the dissociation energy barrier of the crosslinker to 103.0 kj/mol and the energy storage modulus of the hydrogel by 78% while maintaining the intrinsic dynamic properties. Xu et al. [57] prepared HA hydrogels with tunable crosslinking and reversible phase transitions for 3D printing by the dynamic coordination of Fe^3+^ ions with natural carboxyl groups **(**Fig. 1G**)**. By adjusting the concentrations of Fe^3+^ and H* ions and the reaction time, they achieved a low-to-high crosslinking density and reversible solid–liquid phase transition of the HA hydrogels. Li et al. [115] applied HAMA and further introduced GelMA Enzyme-UV2 to creased a double crosslinked hybrid hydrogel (HAMA-P-GelMA). This hybrid hydrogel has a hydrogel matrix with better physical properties (mechanical properties, swelling, and degradation rate) and shows improved cell viability, adhesion, and spreading.

## HA-based bioinks for 3D printing

3D bioprinting, also known as biomanufacturing, is a new additive manufacturing technology for the fabrication of structures that are structurally similar to natural biological tissues. It holds great promise in the fields of regenerative medicine and tissue engineering because of its high precision, controllability, direct cell embedding ability, and good reproducibility of complex structures suitable for restoration [117, 118]. 3D bioprinting uses a series of process and materials (e.g., bioinks), generally in conjunction with computer-aided design (CAD) [119]. Various 3D bioprinting technologies have emerged in response to the demand for complex structures, and they can mimic organs and tissues with high precision. In this section, we elaborate on the principles of 3D bioprinting technologies currently used for HA-based bioinks along with their main advantages and disadvantages.

### Droplet-based bioprinting

**Fig. 2:**
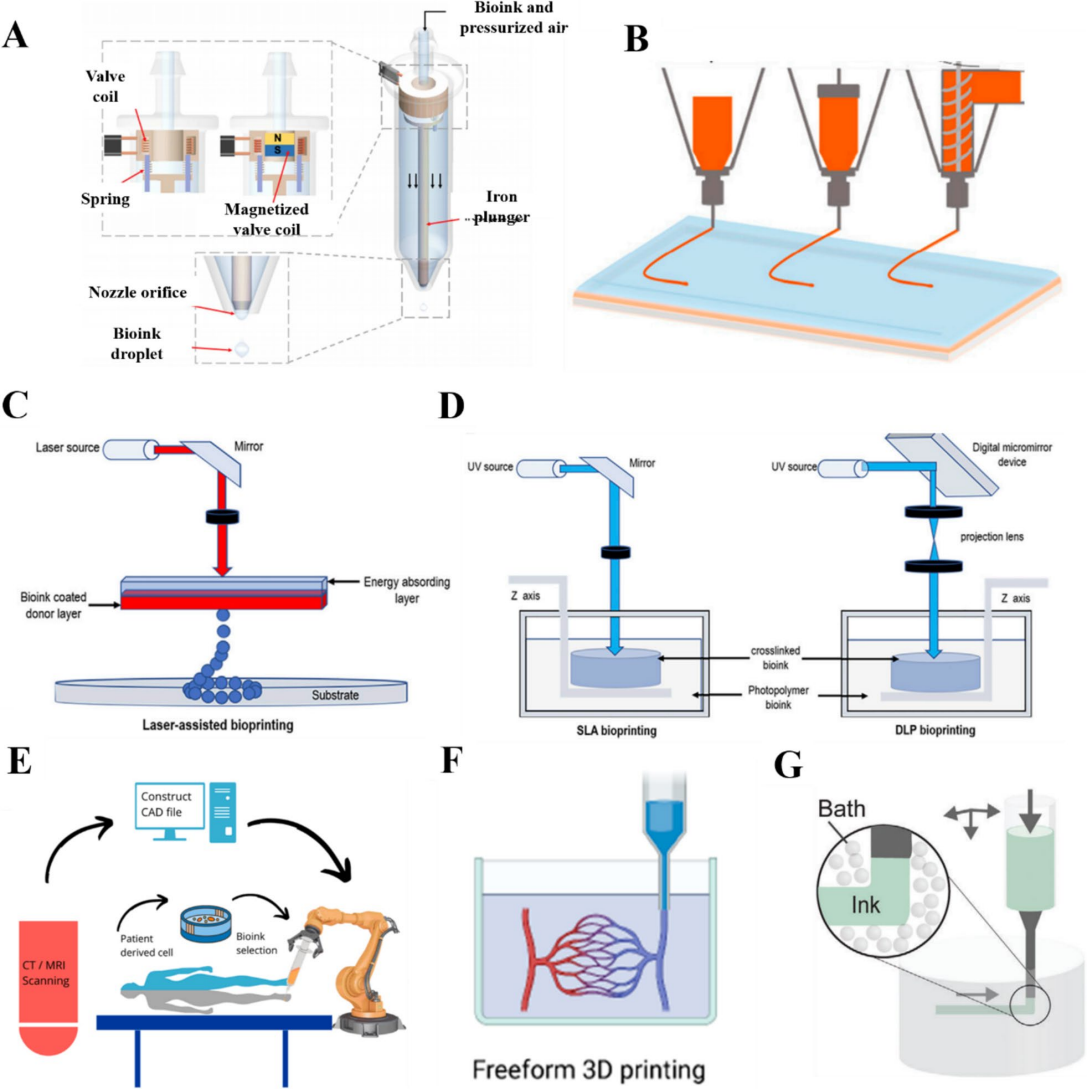
3D bioprinting methods. **A** Droplet-based bioprinting [123]. Copyright 2016, Elsevier Ltd. All rights reserved. License Number: 5618191482149. **B** Extrusion bioprinting [126]. Copyright 2021, the Authors. Licensee MDPI, Basel, Switzerland. Based on Creative Commons Attribution License (CC BY). **C** Laser-assisted bioprinting [127]. Copyright 2022, the Authors. Published by Frontiers. Based on Creative Commons Attribution License (CC BY). **D **Stereolithography (SLA) and digital light processing (DLP) [127]. Copyright 2022, the Authors. Published by Frontiers. Based on Creative Commons Attribution License (CC BY). **E** In situ bioprinting [128]. Copyright 2019, Acta Materialia Inc. Published by Elsevier Ltd. All rights reserved. License Number: 13911336–1. **F** Freeform 3D printing [129]. Copyright 2022, the Authors. Published by American Chemical Society. Based on Creative Commons Attribution License (CC BY). G. Suspension printing [130]. Copyright 2021, Wiley–VCH GmbH. License Number: 5618230459998

Droplet-based bioprinting technologies **(**Fig. 2A**)** print ink droplets in nanometer and micrometer volumes as required for printing [120–122]. The most popular type of droplet bioprinting is inkjet bioprinting, which includes continuous, motorized, and inkjet-on-demand methods. The advantages of droplet-based bioprinting are high cell viability, fast printing speed, low cost, and high stimulability [123, 124]. Droplet printing devices often include a droplet recycler to reduce material waste. However, recycling bioinks can lead to contamination. Unfortunately, pure and unmodified HA bioinks are not suitable for the production of printable bioinks at working concentrations. Aqueous HA solutions have a low viscous modulus, which means they cannot retain their shape or provide adequate yield stress during printing. Therefore, HA is typically mixed with other polymers to produce bioinks for droplet printing [125].

### Extrusion-based bioprinting

Extrusion-based 3D bioprinting technologies** (**Fig. 2B**)** are widely used because of their simplicity, speed, and ability to embed live cells during printing. These printers disperse biological inks using pneumatic or mechanical methods to create 3D structures. The bioink is placed in a printing chamber and pumped through a nozzle combined with a motorized extrusion system. The bioink is extruded from the nozzle tip under the pressure of the motor. A foot pedal can be used to adjust the pressure from the bioink chamber and activate the UV curing device [131–133].

Velasco-Rodriguez et al. [133] used 3D bioprinting to transplant a target kidney organ and modified the kidney organoid by extrusion bioprinting to increase the final number of kidney units from the same starting cell count. Wang et al. [52] developed an extrudable hydrogel bioink for 3D printing based on photocrosslinked GelMA and dynamic hydrazone-crosslinked HA. The hydrogel could be extruded into uniform filaments and printed into scaffolds layer-by-layer owing to its shear-thinning behavior. Shin et al. [134] designed a hydrogel system based on the phenol modification of ECM components (especially HA and gel). This unique gallol-modified ECM ink exhibited transient crosslinking and early shear-thinnin g properties for extrusion-based 3D printing. Palladino [135] proposed a strategy for combining collagen (Col) with tyramine-modified hyaluronic acid (THA) to obtain printable Col-THA bioinks for extrusion bioprinting, and the resulting product has cellular and extracellular matrix anisotropy.

### Laser-assisted bioprinting

Laser-based bioprinting technologies** (**Fig. 2C**)** can build scaffold-free 3D structures based on the directional deposition of printing materials with optical crosslinking by laser irradiation. These techniques include SLA and laser-assisted bioprinting (LAB) [136, 137]. SLA is a layer-by-layer technology that utilizes the photo-selective crosslinking behavior of bioinks **(**Fig. 2D**)**. The photolytic crosslinking of bioinks with a laser or digital light projector allows for a single layer of molecules on the printing plane to be crosslinked, thereby overcoming the transient technical limitations with 3D bioprinting; however, it can cause turbulence, such as high print sharpness [138, 139]. The biotech company Cellbricks developed a stereolithographic bioprinting technique based on multi-material projection, and it offers high printing speeds and spatial resolution [140]. More than 10 years after the advent of SLA, DLP was introduced, which is recognized as a second-generation photocuring molding technology **(**Fig. 2D**)**. It offers high printing resolution and printing speed, enabling the construction of models with relatively high complexity and accuracy. The resolution of LAB matches the size of individual cells, making it a promising tool for simulating the heterogeneity and structural characteristics of skin. It is therefore used in autograft facial surgery by printing simulated skin for plastic surgery [125]. LAB workstations can be set up in sterile operating rooms to provide personalized and “tailor-made” treatment [141].

### Emerging printing technologies

In situ bioprinting** (**Fig. 2E**)** involves the direct printing of bioinks in clinical settings to construct or repair living tissues or organs at defective sites, where the use of ex situ bioprinting techniques is limited [142, 143]. For example, Ma et al. [144] used robot-assisted in situ 3D bioprinting for cartilage regeneration. Researchers have particularly focused on the in situ printing of MSCs with collagen and HA matrices to promote the regeneration of skin and bone injuries [145, 146]. Robot-assisted in situ 3D bioprinting is ideally suitable for improving surgical procedures and promoting cartilage regeneration [144].

Freeform bioprinting, wherein a bioink containing a cell suspension is extruded onto a support material, has attracted considerable attention as a promising bioprinting technology **(**Fig. 2F**)**. Support materials provide physical support during freeform bioprinting, and the gelation of embedded inks in support grooves facilitates the preparation of soft structures with good shape fidelity. Sakai et al. [147] developed an advanced freeform bioprinting technique wherein a bioink containing choline, horseradish peroxidase, and HA derivatives was extruded onto a support material containing choline oxidase. Using this method, they successfully obtained 3D HA-based hydrogel structures with good shape fidelity.

Four-dimensional (4D) bioprinting is an emerging bioprinting technology for the fabrication of loaded cellular structures that can respond to internal cellular forces or external stimuli [148]. 4D bioprinting is similar to 3D bioprinting but uses smart materials that can respond to stimuli, such as water, temperature, pH, electricity, light, ionic strength, magnetic fields, pressure, or sound waves. For example, 4D bioprinted smart “biobots” can be used for disease diagnosis or programmed-release drug delivery. Moreover, smart tissue structures can be realized using this concept for better regeneration of living tissues and organs [149].

Infiltration-induced suspension bioprinting is a novel printing technology that has been proposed to regulate the properties of printed scaffolds through osmosis** (**Fig. 2G**)**. This technology was inspired by the phenomenon in which hydrogels exchange fluids during osmosis. The change in osmotic pressure can guide the contraction or expansion of HA bioinks, thus regulating the physical properties of 3D printed scaffolds, including the mechanical strength, micromorphology, fiber diameter, and water absorptivity [150].

## Applications of HA bioinks

Bioinks are used in 3D printing to print bioscaffolds for tissue engineering. Bioscaffolds offer a suitable microenvironment for seed cell attachment, growth, proliferation, and metabolism, and they gradually degrade, become absorbed by the body, and are eventually replaced by new tissue [151]. The advantage of HA as a scaffold material is that its unique 3D gel structure provides a suitable stereoscopic space for seed cell growth. Scaffolds with a regular shape and smooth surface prevent secondary damage to the implantation area. As a key component of the ECM, HA can coordinate the interaction between bioactive factors and cells, enhance cell attachment and differentiation, and influence the expression of cell surface receptors [152]. HA scaffolds can be combined with bioactive materials and cells to reconstruct the shape, structure, and function of damaged tissues and organs, thereby making them suitable for the replacement of damaged tissues. In addition, HA can be loaded with various molecules and proteins, such as growth factors and cytokines, to further broaden its application scope and enhance its therapeutic effect [153]. Computed tomography data from patients can be used to create tailor-made implant structures. A variety of bioprinting techniques are available to generate tissues and organs based on these data, facilitating the use of HA bioscaffolds in regenerative medicine [154]. This section provides a detailed exploration of some of the emerging applications of HA bioinks in regenerative medicine.

### Cartilage regeneration scaffolds

Recently, the use of autologous cells, biomaterial scaffolds, and growth factors as a potential pathway for repairing cartilage defects has attracted significant interest in the field of regenerative medicine. Currently, the most serious problem with these technologies is the lack of biocompatibility of commonly used polymeric materials [155]. HA induces the production of fibroblasts, keratinocytes, and proinflammatory cytokines, and it also promotes the inflammatory responses of osteoblasts and osteoclasts, which stimulate the synthesis of vascular endothelial cells. This in turn stimulates the regeneration, chemotaxis, proliferation, and differentiation of bone mesenchymal cells, thereby inducing osteogenesis by acting on bone morphogenetic and bridging proteins [156].

**Fig. 3:**
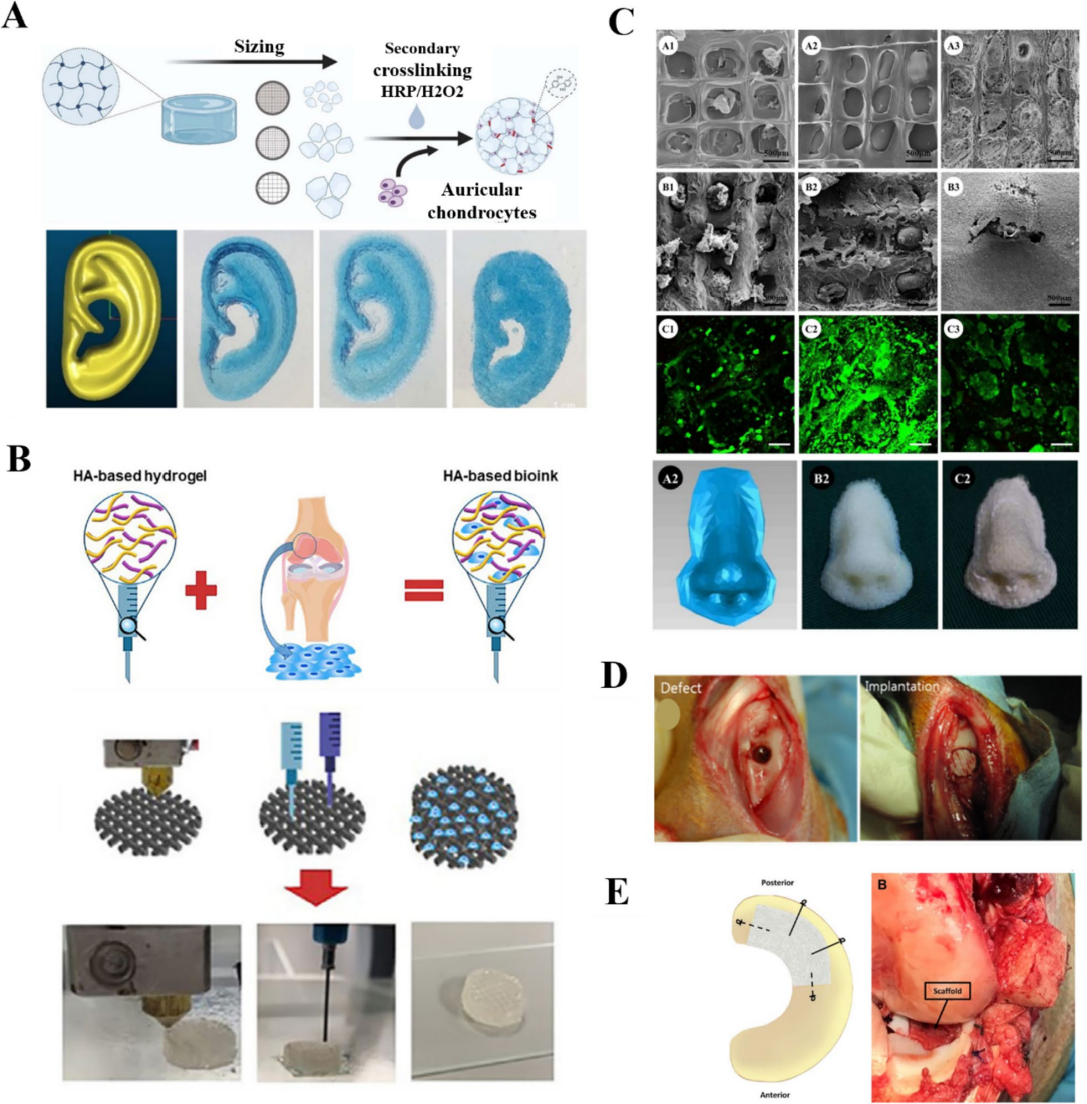
HA for cartilage engineering. **A** HA microgel bioink for ear cartilage printing [59]. Copyright 2022, the Authors. Published by IOP Publishing Ltd. Based on Creative Commons Attribution License (CC BY). **B** HA-based bioink for articular cartilage printing [107]. Copyright 2020, the Authors. Published by Elsevier Ltd on behalf of Acta Materialia Inc. **C** HA-based bioink for nasal cartilage printing. Reprinted (adapted) with permission from [161]. Copyright 2018, American Chemical Society. **D** HA scaffold implanted into rabbit knee joint [162]. Copyright 2016 IOP Publishing Ltd. License Number: 5618230459998. E. Collagen-infused-HA meniscus 3D [163]. Copyright 2019, Wiley Periodicals, Inc. License Number: 5618240771169

Hung et al. [157] developed an HA-based bioink containing synthetic degradable polyurethane elastomeric nanoparticles, HA, and bioactive components. The printed scaffolds provided a suitable matrix for cartilage repair by promoting the self-aggregation of MSCs and releasing bioactive components to induce MSC differentiation into cartilage cells. Shopperly et al. [58] investigated the mixing and layering of HAMA and GelMA inks to simulate the ribbon structure of articular cartilage. The stiffness of the mixed bioink gradually increased with increasing HAMA contents from 2.41 ± 0.58 kPa (5% GelMA, 0% HAMA) to 8.84 ± 0.11 kPa (0% GelMA, 2% HAMA). Flegeau et al. [59] prepared an HA-based bioink with modularity and tunable porosity using granular hydrogels composed of annealed HA microgels **(**Fig. 3A**)**. The HA microgel-printed bioscaffolds were stable in solution and showed adjustable porosity from 9 to 21%. Moreover, the HA-particle hydrogels supported the homogeneous development of mature cartilage-like tissue in vitro. Cultures of printed microgels containing human ear chondrocytes showed progressive maturation of the cartilage tissue. After 63 d, live cells and mechanical sclerosis at up to 200 kPa were achieved. Ku et al. [60] alternately printed polycaprolactone (PCL) and HAMA layers to prepare a scaffold with enhanced biomechanical properties. They then introduced the active factor kartogenin and active material tricalcium phosphate (TCP) into the scaffold. The use of HA improved the scaffold performance, biocompatibility, and cellular activity. Liu et al. synthesized HA- and heparin-based spherical hydrogel particles via inverse emulsion polymerization. The HA-based hydrogel particles induced cartilage formation and triggered the release of growth factor BMP-2 to create an inherent bioactive delivery vehicle [158, 159]. Wang et al. [160] synthesized thiolated heparin and mixed it with HAMA and growth factors, which were then converted to a stable hydrogel via 3D printing. This process allowed the active ingredients to work better, and the prepared HA-based hydrogel scaffold represents an appealing candidate for use in tissue regeneration and ongoing therapy.

Antich et al. [107] prepared HA and Alg-based hydrogel bioinks and co-printed them with polylactic acid (PLA) **(**Fig. 3B**)**. The prepared scaffolds promoted tissue formation by increasing the expression of cartilage gene markers and specific matrix deposition, thereby improving cell function. Mao et al. [164] first loaded transforming growth factor-β1 onto a silk fibroin (SF) scaffold via physical absorption and then coated the scaffold with HAMA, methacrylonylated serine protein, and a marrow MSC-specific affinity peptide. The programmed release of the bioactive molecules promoted in situ cartilage regeneration. Furthermore, microspheres adhered to the joint surface, resulting in targeted drug enrichment near the cartilage. Staubli et al. [165] proposed a THA-Col 1 composite support for the migration and chondrogenic differentiation of human MSC microspheres, and they realized chondrogenic matrix deposition throughout the hydrogel and cell differentiation along the chondrocyte lineage. Yu et al. [166] has designed a new type of photothermal nano-enzymatic material that mimics hyaluronan synthase and antioxidant enzyme activities to regulate catabolism and anabolism in the treatment of osteoarthritis, thereby attacking the problem that "cartilage is difficult to repair once it has been damaged.

However, new tissue growth does not integrate well with HA. Therefore, for cartilage repair, HA is commonly loaded with cells and used as a carrier. Alternatively, the addition of active factors can promote cartilage ECM synthesis and cell differentiation. HA plays an important role in various tissues, including articular cartilage, ear cartilage, nasal cartilage **(**Fig. 3C**)**. The use of HA alone to repair osteochondral defects has some beneficial effects** (**Fig. 3D**)**. In addition, it plays a role in the repair of the meniscus in articular cartilage** (**Fig. 3E**)**.

### Bone tissue engineering

The conventional approach to bone repair is autologous bone grafting; however, owing to the limited number of autologous bone donors, risk of rejection, and donor complications, alternative methods are needed for the repair of large segmental bone defects. Bone tissue-engineered scaffolds are considered ideal alternatives to autologous bone grafts owing to their biocompatibility [167]. HA, as one of the main components of the ECM, can produce the necessary extracellular mass in an appropriate manner. Eventually, healthy bone tissue with acceptable geometry, composition, and size is formed to reconstruct the cellular microenvironment and rebuild the entire organ [168]. HA-based hydrogels are well-suited to the construction of 3D geometries by micro-extrusion bioprinting. In addition, they are suitable substrates for bone matrix development and remodeling. This makes HA-based composite hydrogels excellent materials for bone tissue engineering [169].

**Fig. 4:**
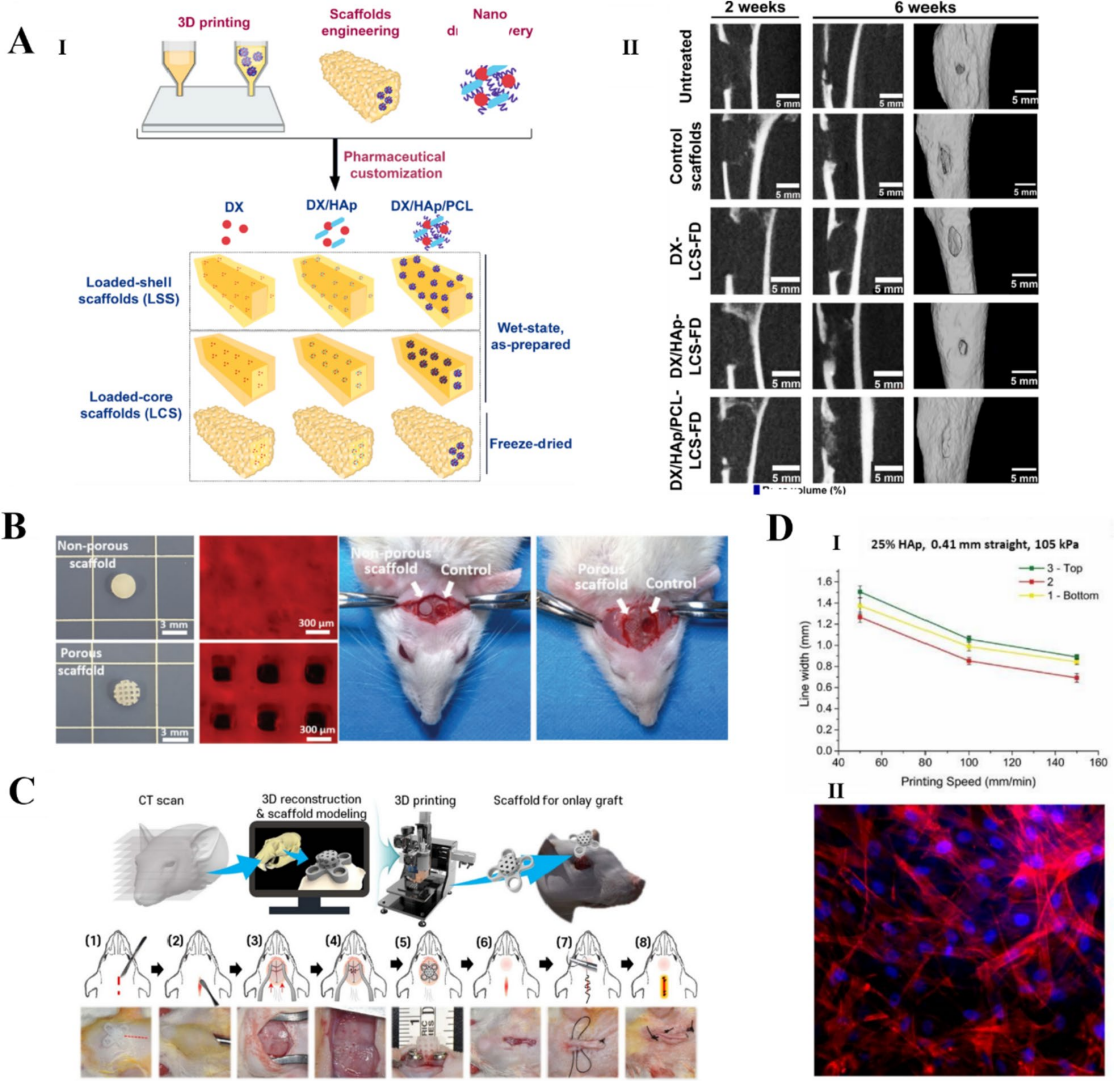
HA for bone tissue engineering. **A** (I) Loaded-core scaffold (LCS) with a polycaprolactone (PCL) ink as the shell phase and drug-loaded integrated doxycycline (DX) nanoparticle ink as the core phase. (II) Cone-beam computed tomography images of bone regeneration in tibial samples showing the superiority of DX/HAp/PCL-LCS freeze-dried scaffolds for in vivo bone regeneration [170]. Copyright 2021, Elsevier B.V. All rights reserved. License Number: 5619340180570. B. Cranial bone regeneration after 4 and 8 weeks of in vivo placement of NIDN hydrogel scaffolds [171]. Copyright 2021, Wiley–VCH GmbH. License Number: 5619340843709.** C** Implantation of rat cranial high inserts integrated into the recipient bone [60]. Copyright 2021, the Author(s). Published by IOP Publishing Ltd. Based on Creative Commons Attribution License (CC BY) **D**. 3D printing bioink for bone tissue engineering. (I) Printing speed vs. line width using a 25% HAp ink, 0.41 mm straight steel needle, and 105 kPa printing pressure. (II) Adhesion of 25% HAp ink surface after 3 days of incubation with MSCs in spindle-like morphology [172]. Copyright 2019, Elsevier B.V. All rights reserved. License Number: 5619341400346

Wenz et al. [168] produced polymer solutions based on GelMA and HAp particles modified with HAMA. Primary human adipose-derived stem cells were encapsulated in a gel containing HAp particles and cultured for 28 d. The additional use of osteogenic media resulted in a 199% ± 27.8% increase in storage modules. Wei et al. [167] developed a composite bioink consisting of SF, GEL, HA, and TCP. 3D printed composite scaffolds were combined with platelet-rich plasma post-treatment, which significantly promoted the growth and proliferation of MSCs. Yang et al. [169] produced an osteoblast hydrogel for use in a 3D bioprinting bioink that consists of GelMA, HAMA, and type I Col. The scaffold had excellent shape fidelity and exhibited high cell viability (85–90%) and cell density (10 cells/mL) during in vitro biomimetic mineralization. Moreover, the bioactivity of the HA polymer matrix and the osteogenic properties of the bound bioactive nanoparticles acted synergistically to enhance bone regeneration in vivo without compromising biodegradability. El-Habashy et al. [170] developed an active HAp/PCL nanoparticle hydrogel scaffold that was osteoconductive, biodegradable, and biocompatible** (**Fig. 4A**)**. It was successfully used for bone healing in a rabbit tibial bone model. A knitted mesh containing HA benzyl ester was used to cover the bone defect in the rabbit model, which acted as an effective allograft and aided in the restoration of the periosteal connective tissue.

Pure HA bioink has insufficient mechanical properties for printing high-strength scaffolds, such as bone tissue. Therefore, nanoclays have been used to improve the physical and mechanical properties of printed scaffolds, either by mixing the nanoclay directly with the bioink or by coating the nanoclay on surface of the scaffold** (**Fig. 4B**)**. Lim, Kang, & Jeong [173] prepared nanocomposite hydrogels suitable for 3D bioprinting by adding nanodiamonds to crosslinked HAMA. The HA composite hydrogel with 0.02 wt% nanodiamonds had an improved compressive strength and gel rupture point. Roushangar Zineh et al. [174] designed a novel biomaterial composed of Alg, HA, kaolinite nanotubes, and polyvinylidene fluoride. Experimental and numerical analyses showed that the kaolinite nanotubes increased the tensile and compressive strengths of the composite by 47%. This provided the mechanical strength required to prepare an efficient biological scaffold but did not compromise the biological properties. Kim et al. [62] combined bionanotechnology with 3D printing to prepare biomineralized gel/HA/HAp composite bone tissue scaffolds with adjustable compositions and morphologies. They prepared bilayer composite scaffolds with both interlaced orthotropic and alternating structures. The microstructure of the scaffolds mimicked the structure of the ECM, with a wrinkled inner surface and porous hierarchical structure, which effectively promoted cell proliferation and osteogenic differentiation. Farsi et al. [175] used fused-deposition modeling to prepare PLA scaffolds for cartilage applications and then electrostatically spin-coated the scaffolds with polyvinyl alcohol (PVA) and HA fibers. The elastic moduli of the PVA/PLA and PLA/PVA/HA scaffolds were increased to 18.31 ± 0.29 and 19.25 ± 0.38 MPa, respectively, by electrostatic spin-coating. Hydrophilic HA covered the surface of the PLA scaffold, thereby reducing the contact angle and improving its hydrophilicity. In addition, HA increased the molecular polarity of the scaffold complex because of its hydroxyl, amine, and carboxyl groups. Garnica-Galvez [176] evaluates the physicochemical properties and biological consequences of mesenchymal stromal cell cultures of single and mixed HA molecules. It was found that in HA in type III collagen deposition was more pronounced and induced a higher degree of mineralization that enhanced chondrogenesis and osteogenesis. Tao et al. [63] designed an HA/poly-l-lysine (PLL) layered self-assembled coating on a β-TCP scaffold, which provided a modular system based on the fixation of small extracellular vesicles with c(RGDfC), a highly effective peptide targeting αvβ3, surface functionalization and ZEB1 loading. The scaffold facilitated the regeneration of bone defects in diabetic conditions. HA is a negatively charged glycosaminoglycan, and the interaction between positive and negative charges helps HA anchor to the binding sites of surface proteins (e.g., CD63, CD81, and CD9). After surface functionalization of c(RGDfC), SEV-carrying ZEB1 enhances osteogenic differentiation by enhancing downstream genes of YAP through ZEB1-YAP interactions in an in vitro medium and enhancing bone formation in vivo in a diabetic rat model of cranial bone defect. HA as a bio-ink can be used to repair large bone defects by 3D printing** (**Fig. 4C**)**, which could increase cell activity and facilitate proliferation** (**Fig. 4D**)**.

### Skeleton muscle tissue engineering (SMTE)

SMTE aims to repair defects and rebuild the structure and function of skeletal muscle, providing a novel option for clinical treatments for muscle tissue repair and regeneration. One of the current goals of SMTE is to develop methods of producing bionic and functional structures [177], which could provide an alternative to current treatments for volumetric muscle loss, such as prosthetic supports and autologous muscle flap grafts. 3D bioprinting is uniquely suited to SMTE because it can mimic the complex microstructures of tissues and precisely control the deposition and cell alignment of cellular materials. The arrangement of myogenic cells is crucial for the engineering of muscle formation and anisotropic skeletal muscle tissue [178].

**Fig. 5:**
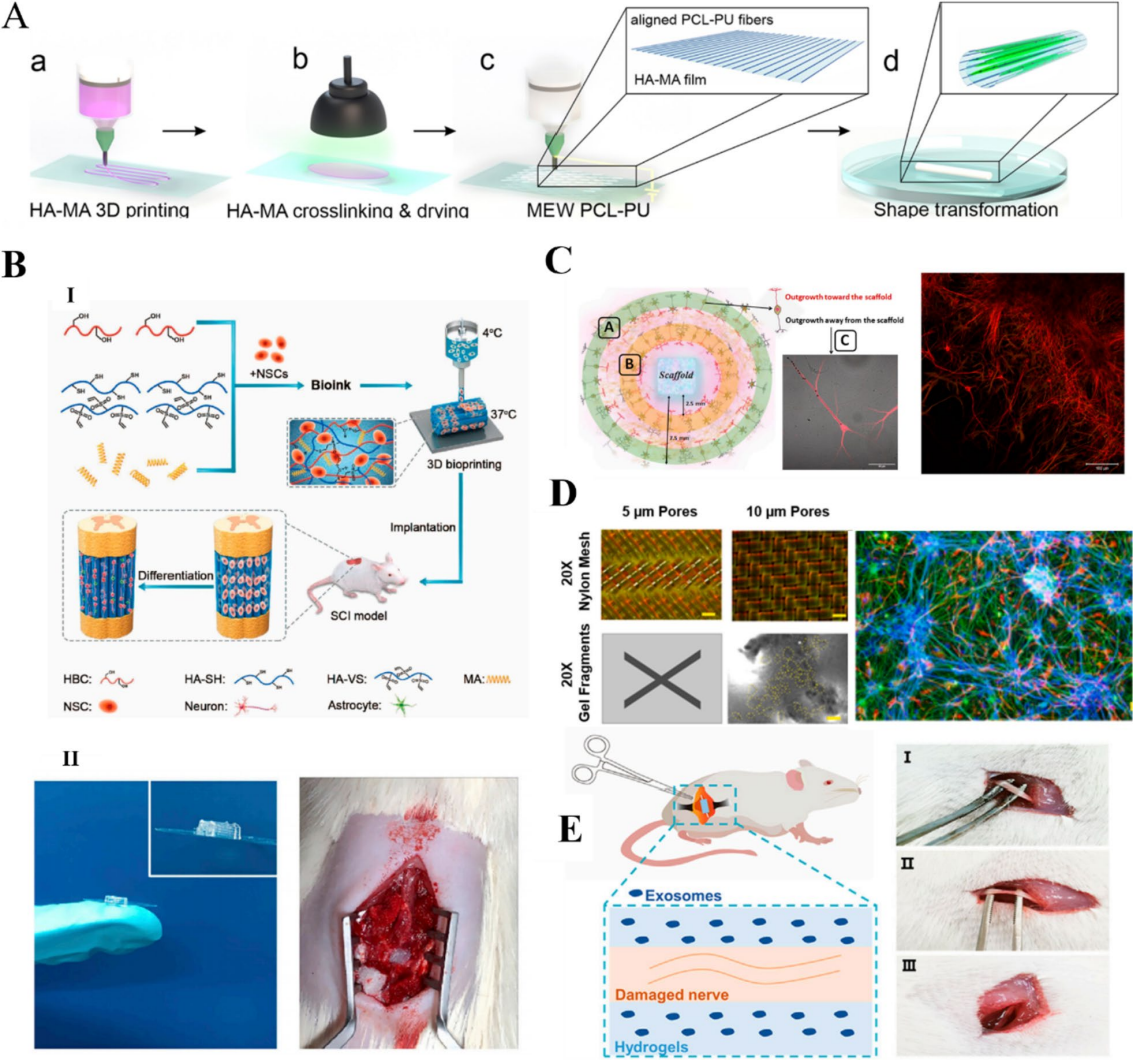
HA for skeletal muscle tissue engineering (SMTE) and neural engineering. **A** 3D printing of HAMA for SMTE. Reprinted (adapted) with permission from [64]. Copyright 2021, American Chemical Society. **B** 3D bioprinted neural tissue scaffold for in vivo spinal cord injury repair. (I) Schematic of neural stem cell (NSC)-loaded bioprinted scaffold. (II) Photographs of 3D bioprinted neural tissue scaffold and implantation into the lesion area of spinal cord injury rat model [65]. Copyright 2021, Elsevier Ltd. All rights reserved. License Number: 5620791313738. **C** Perfusion of neurotrophic factors (BDNF, 20% GDNF) in 3D printed HA scaffold with a large number of interconnections between neurons and astrocytes [180]. Copyright 2022, the Authors. Published by Frontiers. Based on Creative Commons Attribution License (CC BY). **D** Neural precursor cells cultured in HA-based hydrogel composite for 3 months with well-aligned HIPSC-derived neural clusters surrounded by astrocytes [181]. Copyright 2022, Elsevier B.V. All rights reserved. **E**. HA hydrogel-loaded exosome treatment for sciatic nerve injury in rat model [182]. Copyright 2022, the Authors. Published by Frontiers. Based on Creative Commons Attribution License (CC BY)

Co-culturing after the differentiation of cells or the induction of pluripotent stem cells is commonly performed to promote the formation of vascular networks and neuromuscular junctions. In addition, to restore the function of the skeletal muscle tissue, vascular and neural networks are required for material exchange [179]. In vivo skeletal muscle vascularization can be achieved by stimulating the inward growth of existing vessels to form new capillaries. As a component of the ECM, HA plays a role in SMTE through cell migration, proliferation, and differentiation. Uribe-Gomez et al. [64] reported an HA-based 3D printed hydrogel for use in SMTE. The scaffold was formed by 3D printing HAMA followed by the deposition of PCL-PU with melt electro-writing to form uniaxial microfibers **(**Fig. 5A**)**. The scaffold exhibited a good morphology, mechanical properties, and surface chemistry and could support a high degree of myocyte alignment. Next, we investigated the rheological properties and printing of HAMA. Our findings showed that the storage modulus of 3% HA-MA ink for 3D printing was ~ 0.8 Pa at 0.1 Hz and ~ 122 Pa at 100 Hz. After exposure to green light, the storage modulus increased to ~ 5 Pa (0.1 Hz) and 1800 Pa (100 Hz), indicating crosslinking of the polymer. The decrease in storage modulus with decreasing frequency indicates the presence of temporary physical crosslinking, which contributes to the rigidity of the hydrogel at high frequencies. Zhou et al. [51] developed a new bioink for SMTE based on catechol-modified HA and Alg functionalization. The bioink was used to prepare a printed scaffold with high cell viability and the ability to support and guide cell differentiation into aligned myotubes.

### Neural tissue scaffolds

The central nervous system is extremely complex, making it difficult to mimic its physiological structure, particularly when using conventional methods. This has traditionally limited the therapeutic effects of treatments for neural tissue injuries. Cell therapy for CNS disorders usually requires large numbers of cells, and therefore, high-throughput methods must be developed to generate these cells. Although direct transplantation of cells in the damaged CNS is possible, these cells often do not integrate properly into the brain [183]. In contrast, 3D bioprinting technologies provide a novel strategy for neural tissue repair due to the development of neural tissue scaffolds with specific biological and physical functions [184]. The implanted scaffolds provide mechanical support to the spinal cord tissue, guide nerve cell growth, improve the microenvironment at the injury site, and promote spinal cord repair [185]. Liu et al. [186] produced a scaffold loaded with neural stem cells (NSCs) using extrusion-based 3D bioprinting. The scaffold enabled the implanted NSCs to survive for up to 12 weeks in vivo. Moreover, after implantation into spinal cord-injured rats, the implanted NSCs differentiated into neurons, formed nerve fibers, and achieved axonal regeneration, which notably improved the motor function in the hindlimbs of the rats **(**Fig. 5B**)**.

Despite the above-mentioned advances, it is difficult for bionic scaffolds to satisfy the requirements for spinal nerve electrical signal transmission. To address this, Yuan et al. [127] developed a novel conductive hydrogel based on HAMA, GelMA, and poly(3,4-ethylenedioxythiophene): lignin sulfonate. HAMA and GelMA, which mimic the ECM of the nervous system, provided mechanical support for the scaffold and a suitable growth environment for NSCs. The mechanical properties of the hydrogel were similar to those of spinal cord tissue (energy storage modulus of approximately 1 kPa), and its porous structure and solubilization properties were suitable for NSC growth. Samanta et al. [187] covalently grafted dopamine fractions onto hydrazone-crosslinked HA–chondroitin sulfate composite hydrogels to form a 3D scaffold that mimicked brain function and brain structure and promoted neuronal network formation. The composite hydrogel supported the growth of neuronal protrusions and promoted the maturation of nerve cells, resulting in remarkable synaptic growth.

Brain tissue reconstruction after a traumatic brain injury remains a long-standing challenge in neuro transplantation. Mishchenko et al. [180] impregnated 3D HA scaffolds with neurotrophic factors (BDNF, GDNF)** (**Fig. 5C**)**. The scaffolds had a Young’s modulus of 600 kPa, swelling rate of 336%, and no significant cytotoxicity. Engineered biomaterial microenvironment can help overcome low cell survival after damaged CNS transplantation and limit cell migration from the implantation site while providing a controlled environment for cell growth and differentiation [183] **(**Fig. 5D**)**. Shi et al. [66] prepared phenylboronic acid modified hyaluronic acid (HA-PBA)/PVA dynamic hydrogel-coated neural precursor cells that showed good viability in vitro. The hydrogel coating protected the neural precursor cells from damage by reactive oxygen species (ROS) when H_2_O_2_ was present in the culture medium. Owing to their good histocompatibility and adjustable mechanical properties, HAMA hydrogels loaded with exosomes are suitable for the repair of sciatic nerve injuries. Exosome-loaded soft hydrogels are particularly effective for repairing injured peripheral nerves **(**Fig. 5E**)**. Notably, they inhibit macrophage infiltration by rapidly releasing exosomes that restrain the expression of proinflammatory factors, such as IL-1β and TNF-α, in damaged nerves while promoting nerve repair [182].

### Skin tissue engineering

The skin plays a crucial role in protecting the body from the external environment and exogenous stress. However, it is vulnerable to injuries, such as wounds and burns. Skin wound healing requires the closure and repair of skin defects, which involves a complex and dynamic set of biological processes [188]. In the early stages of the wound healing process, activated platelets produce large amounts of high-molecular-weight HA, which promotes coagulation mechanisms by enhancing fibrinogen deposition, and inflammatory cytokines at the wound site are involved in the splitting of high-molecular-weight HA to low-molecular-weight HA [189–191]. Leukocyte and monocyte migration is facilitated by HA-CD44 interactions, and tumor necrosis factor (TNF) and interleukins (IL-6 and IL-1) are secreted by HA-oll-like receptor (TLR4 and TLR2) interactions. Due to these skin-specific features and functions, different forms of HA-based scaffolds, such as hydrogels, dermal fillers, intradermal injections, and thin films, are used to treat damaged skin. In normal cases, based on the self-healing properties of the skin, wound repair can be achieved simply by keeping the skin clean and preventing infection. However, chronic wounds and large injuries can be more difficult to heal [192]. Exogenous HA plays three main roles in the healing process. First, it forms clots with blood fibrin, which plays a constructive role in the wound healing process. Second, it regulates the inflammatory response by promoting the phagocytic activity of granulocytes. Finally, HA regulates collagen synthesis, which is beneficial for wound healing and reduces scar formation [193]. Owing to their high-water absorptivity, HA hydrogels also provide moisture to the wound and maintain a moist environment for cell migration. HA hydrogels also have high oxygen permeability, good biodegradability, and biocompatibility, which makes them suitable for use as skin substitutes to reduce the instance of microbial infections and shorten the regeneration process [194]. 3D bioprinting technologies can be used to create personalized patches that are tailored to the shape of the damaged tissue. Moreover, these patches can be infused with active substances to improve the healing behavior [9].

**Fig. 6:**
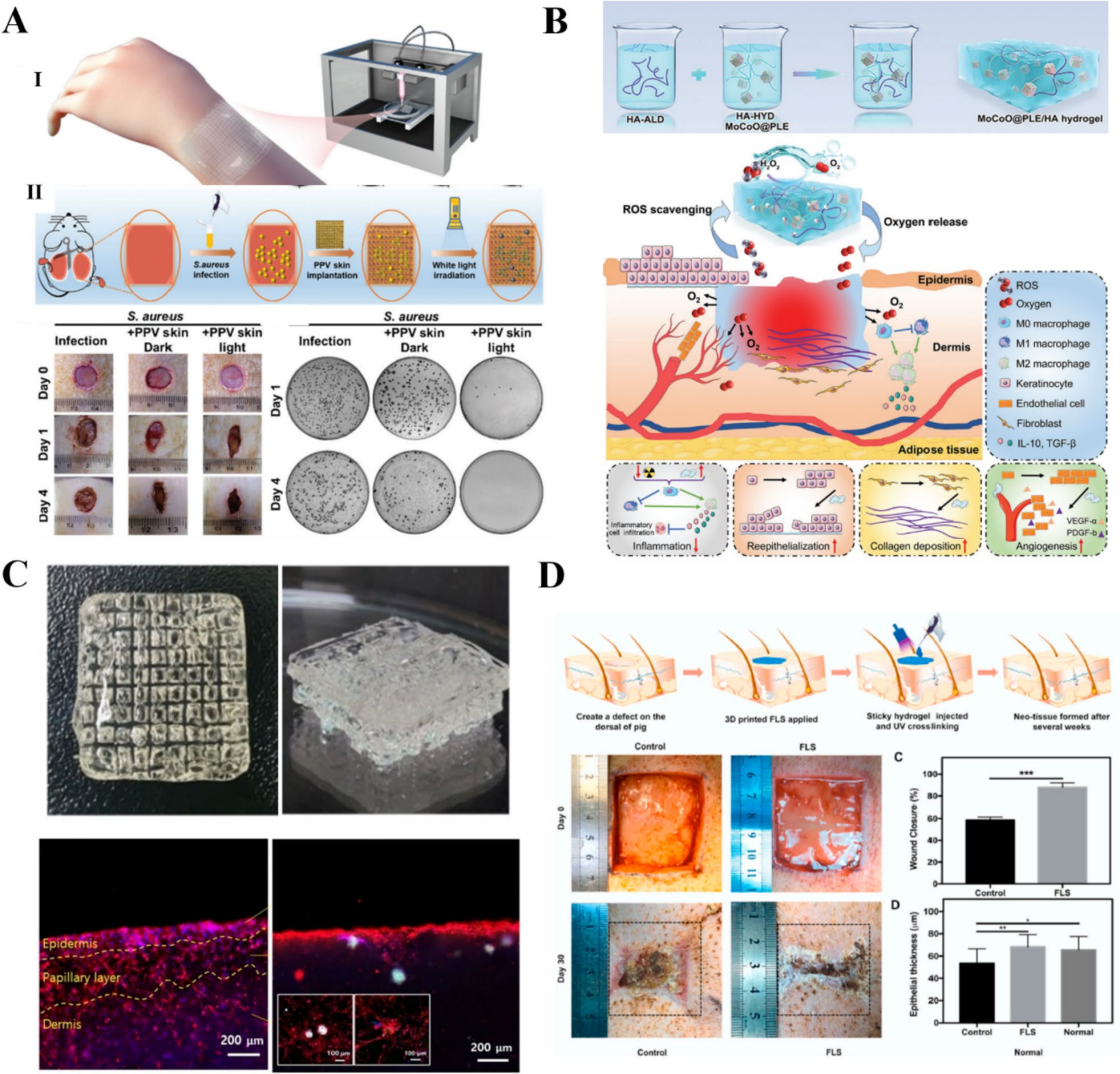
HA for skin tissue engineering. **A** 3D printed Alg/HA/a photoactive cationic conjugated poly (phenylene vinylene) derivative (PPV) skin patch with high cell affinity and antimicrobial properties for post-trauma repair. (I) Schematic diagram of the skin patch. (II) Infected trauma rat model to study the antimicrobial effect of the skin patch [195]. Copyright 2022, Royal Society of Chemistry. License number: 1392769–1. **B** MnCoO@PLE/HA hydrogel patch that accelerates wound healing through continuous reactive oxygen species (ROS) scavenging and oxygen generation [197]. Copyright 2022, Wiley–VCH GmbH. License Number:5620810207094. **C** GelMA/HAMA-based 3D printed skin equivalents containing hair follicles and epidermal/papillary dermal layers [67]. Copyright 2022, Wiley–VCH GmbH. License Number: 5620810653158. **D** Development of a FLS 3D printing technology based on biomimetic GelMA/HANB/LAP bioink that promotes wound healing [141]. Copyright 2020, Elsevier Ltd. All rights reserved. License Number: 5620811275327

Guan et al. [132] used GelMA and HAMA to make 3D bioprinted artificial skin patches. To improve the angiogenic properties, pro-angiogenic QHREDGS peptides were covalently bound to the patches for prolonged release. Flegeau et al. [59] developed 3D bioprinted functional artificial skin patches from a conjugated polymer ink comprising a photoactive cationic conjugated polyderivative and GelMA/Alg/HA. The patches exhibited anti-infective effects based on photodynamic therapy and outstanding bioactivity for wound repair. In addition, Zhao et al. [195] used A5G81, a laminin-derived peptide, to covalently modify artificial skin patches **(**Fig. 6A**)**. The patches had good biocompatibility and exhibited cell migration and adhesion-promoting effects, thus promoting wound healing in vivo. Zhou et al. [196] mixed fibrinogen with human dermal fibroblasts and integrated them with printed scaffolds to induce gelation. The printed scaffolds had high elasticity and supported the formation of bilayer cell-loaded skin-like HA structures based on their ECM-like properties. Such bioactive scaffolds offer new opportunities for skin tissue engineering, especially for the manufacture of skin substitutes with antimicrobial effects. Li et al. [197] found that MnCoO@PLE/HA hydrogel patches accelerate wound healing through continuous ROS scavenging and oxygen generation **(**Fig. 6B**)**. Kang et al. [67] created GelMA/HAMA-based 3D printed skin equivalents containing hair follicles and epidermal/papillary dermal layers **(**Fig. 6C**)**. GelMA/HAMA hydrogels promote epithelial-mesenchymal interactions. In addition, cytocompatibility is observed for the cells loaded in skin equivalents. Nevertheless, the HA content in GelMA/HAMA hydrogels is thought to establish proper cell–cell contact and signaling during development in vitro. Ming et al. [198] encapsulated *Lactobacillus royi* into hydrogel microspheres and used them to prepare a hydrogel dressing in situ by the covalent crosslinking of HAMA. The active probiotic antimicrobial agent inhibited the growth of pathogenic bacteria, resulting in an increased deposition of regenerative collagen and hair follicles in the wound, fewer inflammatory cells, and superior wound-healing ability. Zhou et al. [141] developed a functional living skin 3D printing technology based on biomimetic GelMA/HANB/LAP (photo-initiator lithium phenyl-2,4,6-trimethylbenzoylphosphinate) bioink that promotes wound healing **(**Fig. 6D**)**. Qi et al. [199] prepared a GHM3 hydrogel for the treatment of wounds, which used phenylboronic acid (glucose-responsive), double-bond-modified gelatin, and HASH to encapsulate gold-platinum alloy-deposited melanin AuPt@melanin nanoparticles in order to better adapt to the wound microenvironment. GHM3 effectively alleviated excessive ROS levels in the high-glucose microenvironment and induced M2-type macrophage polarization, thereby reducing inflammatory responses and promoting better wound healing. Wang et al. [200] has developed a macroporous HA hydrogel skeleton hydrogel (DA7CG@C) loaded with the multifunctional antimicrobial peptide DP7 and placental mesenchymal stem cells (PMSCs). DP7 cooperates with the stem cells within the gel to inhibit bacterial proliferation and promote epidermal cell migration and angiogenesis through the secretion of different cytokines at different stages of wound healing.

### Dental materials

**Fig. 7:**
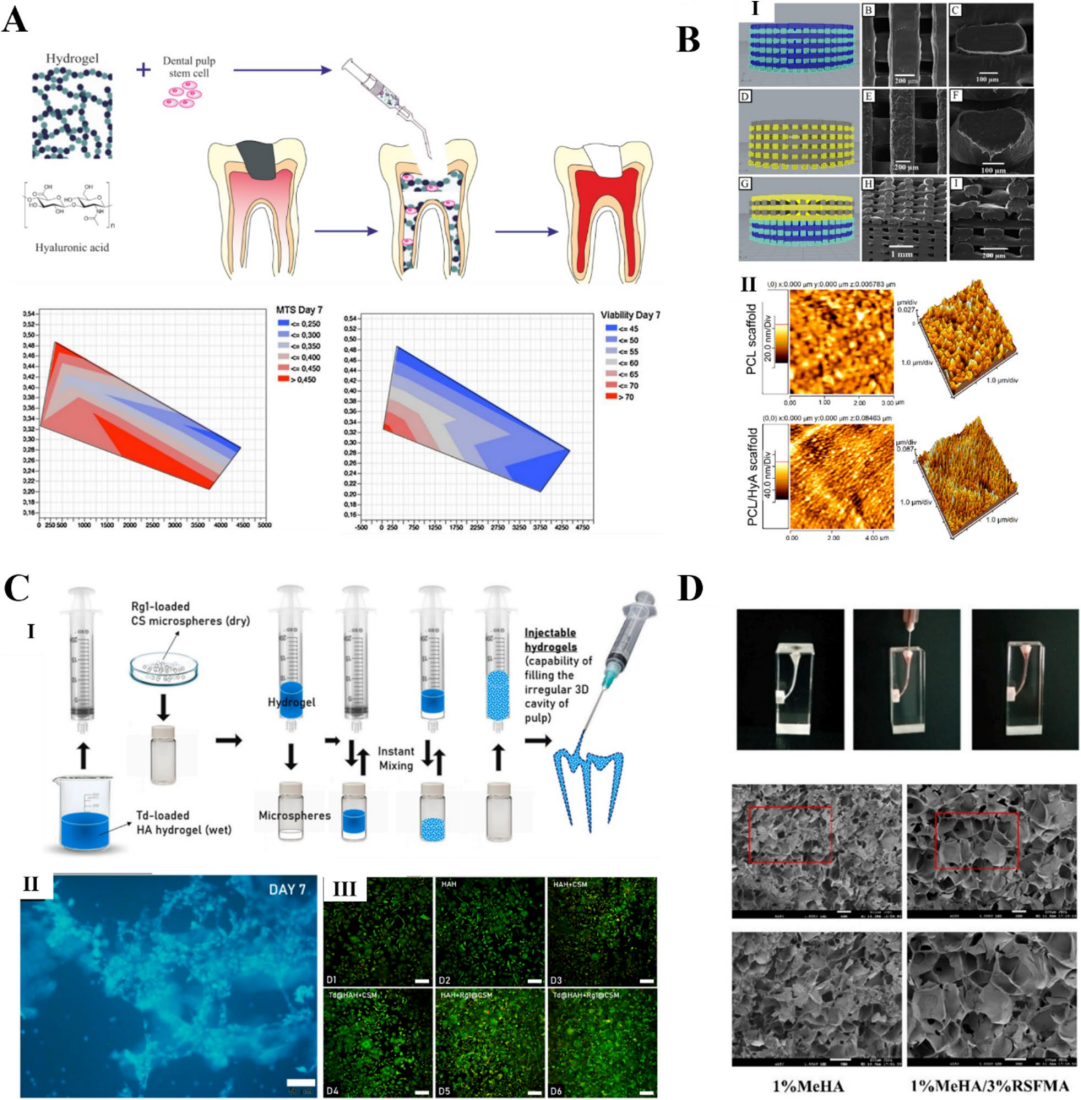
HA for dental materials. **A** HA hydrogel-supported stem cells for the repair of dentin and pulp injuries, and isometric map of apical papilla stem cells showing the advanced metabolic activity of Alg/HA-based hydrogels [201]. Copyright 2019, Elsevier B.V. All rights reserved. License Number: 5620820355951. **B** CAD models and electron micrographs of 3D printed PCL/BG and PCL/HyA bilayer scaffolds for pulp and dentin regeneration [68]. Copyright 2021, the Authors. Licensee MDPI, Basel, Switzerland. Based on Creative Commons Attribution License (CC BY). **C** HA hydrogels containing Rg1-loaded chitosan microspheres, which facilitate cell adhesion and biomineralization during pulp regeneration [202]. Copyright 2021, Elsevier Ltd. License Number:5620821005661. **D** Biocompatible RSFMA/HAMA composite hydrogel, which supports the proliferation and differentiation of human dental pulp stem cells. The addition of RSFMA improves the regularity of the pore size and the mechanical properties of the scaffold [69]. Copyright 2022, Elsevier B.V. License Number: 5620821448137

Owing to the complex and irregular anatomy of the root canal system, the construction of bioscaffolds suitable for pulp regeneration is a significant challenge in dentistry **(**Fig. 7A**)**. Injectable hydrogels have therefore attracted much attention as cell carriers in the field of pulp regeneration. For example, polycaprolactone (PCL)/45S5 Bioglass (BG) composite and PCL/HA (HA) scaffolds were developed by Mousavi Nejad [68], and they promoted the adhesion of human dental pulp stem cells and differentiation of dentin, resulting in a significant increase in the expression of dentin salivary phosphoprotein, osteocalcin, and dentin matrix protein 1, which are markers of dentinogenesis** (**Fig. 7B**)**. Ahmadian et al. [201] noted that HA-based hydrogels are beneficial for maintaining the bioactivity, proliferation, and migration capacity of human dental pulp stem cells and accelerating the bone repair process after tooth extraction. In an in vivo rat tooth defect model, HA-based sponge scaffolds were found to be more effective for the regenerative repair of damaged dentin than collagen. Atila et al. [202] combined injectable HA hydrogel microspheres with Tideglusib (Td) and Rg1 chitosan for vital pulp regeneration **(**Fig. 7C**)**. The microspheres released Td and Rg1 to trigger hDPSCs differentiation and pulp vascularization, respectively. The optimal concentrations of Td and Rg1 were 90 μg/mL and 50 μg/mL, respectively. Wang et al. [69] engineered photocrosslinked RSFMA/HAMA composite hydrogels **(**Fig. 7D**)**, and the hydrogel system was able to fill complex root canal systems under UV light irradiation by spatiotemporal control of the hydrogel network. In addition, the RSFMA/HAMA composite hydrogel exhibited low cytotoxicity and effectively promoted the proliferation and differentiation of dental pulp stem cells (hDPSCs). Wu et al. [203] confirmed the accelerating role of HA in collagen hydrogels and dentin remineralization in vitro. HA provides additional nucleation sites and shortens the induction time of amorphous calcium phosphate (ACP)-mediated hydroxyapatite (HAP) crystallization, which facilitates mineralization. HA modification enhances the calcium ion (Ca2 +) binding capacity by decreasing the electronegativity of collagen surfaces, which produces locally higher supersaturation, resulting in a significant promotion of intrafibrillar collagen mineralization. Biocompatible hydrogels containing proteins and glycosaminoglycans can mimic the chemical and structural characteristics of human soft tissues to assist in soft tissue repair. Although the potential of injectable HA-based hydrogels to improve pulp regeneration has attracted considerable attention, novel hydrogels must be further developed and their feasibility must be evaluated before they can be used in clinical applications.

### Disease models

Organoids, especially patient-derived organoids, have emerged as critical tools in disease research. HA can be used in the construction of organoid disease models to promote drug screening and the development of new methods of diagnosing and treating diseases. Wang et al. [71] developed 3D printed islet-like organs from HAMA/pancreatic ECM hydrogel bioinks. The hydrogels exhibited islet cell morphology and adhesion through the Rac1/ROCK/MLCK (genes) signaling pathway, which contributed to improved islet function and activity. This approach could be used in clinical applications to improve the efficacy and safety of islet transplantation. Zhou et al. [204] improved the mechanical properties, network homogeneity, print resolution, and ROS accumulation during polymerization by developing photosensitive bioinks based on mercapto-norbornene and polysaccharides. The printed liver model showed high albumin secretion and urea production and good sensitivity to drug-induced hepatotoxicity. Van der Valk et al. [205] prepared a 3D bioprinted model of aortic valve calcification using a GelMA/HAMA hydrogel to replicate specific mechanical properties, followed by the 3D printed deposition of encapsulated human valvular interstitial cells. This method provided insights into the mechanobiology of the valve and is expected to facilitate high-throughput drug screening for diseases.

**Fig. 8:**
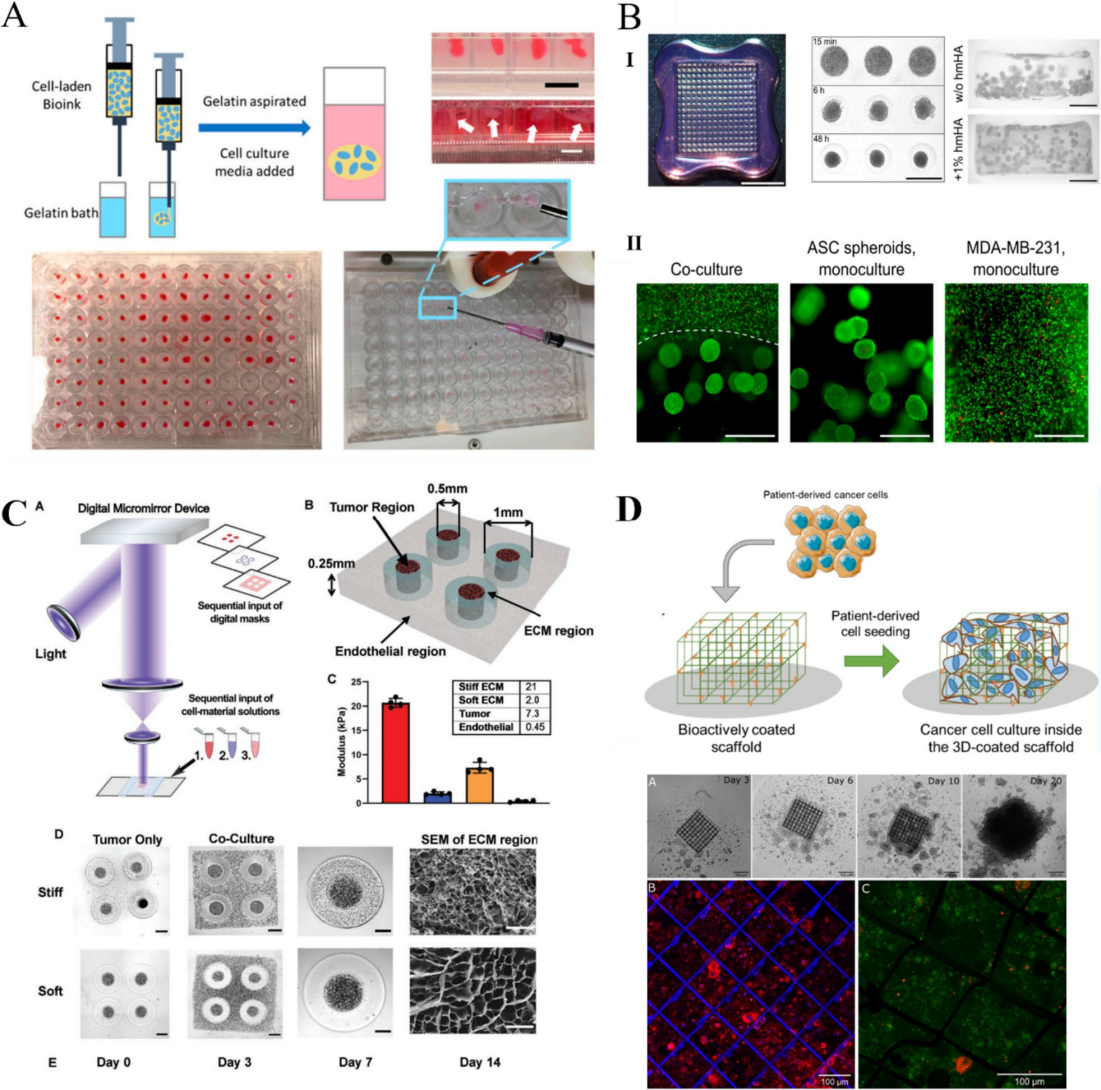
HA for disease models. **A** Immersion bioprinting of tumor tissue-like organs increases the throughput of multi-well plate screening for chemotherapy [206]. Copyright 2021, the Authors. Licensee MDPI, Basel, Switzerland. Based on Creative Commons Attribution License (CC BY). **B** 3D bioprinted breast cancer–adipose tissue model. (I) Printed gel spheroids with 1 wt% high-molecular weight HA to ensure a uniform HA distribution. (II) The printed HA spheroids were subjected to lipogenic differentiation for 21 days with good survival of seed cells [207]. Copyright 2021, the Authors. Licensee MDPI, Basel, Switzerland. Based on Creative Commons Attribution License (CC BY) **C**. 3D bioprinted glioblastoma models consisting of HAMA and (GelMA) with brain tumor-specific ECM-derived bioinks were created for four different tumors [208]. Copyright 2021, Wiley–VCH GmbH. License Number:5620830466879. **D** Two-photon polymerization technology for fabrication of 3D printed microscopic scaffolds to form miniature tumor tissue and mimic metastatic cancer models. Reprinted (adapted) with permission from [209]. Copyright 2022, American Chemical Society

Clark et al. [70] developed a tumor-like organoid immersion model for drug screening via the bioprinting of HA-based hydrogel bioinks. The organoid specimens were derived from glioma and lung adenocarcinoma brain metastases. Maloney et al. [206] developed a 3D printed breast cancer model for studying cancer cell-adipocyte interactions **(**Fig. 8A**)**. The extrusion bioprinting process was optimized based on the sphere viability and uniform HA bioink distribution. The authors noted that 3D printed adipose tissue models of breast cancer can outline important aspects of complex cell–cell and cell–matrix interactions in the tumor stromal microenvironment. Horder et al. [207] printed gel spheroids with 1 wt% high-molecular weight HA to ensure a uniform HA distribution with good survival of seed cells **(**Fig. 8B**)**. Tang et al. [208] used 3D bioprinting technology to achieve rapid, flexible, and reproducible glioblastoma multiforme (GBM) modeling and developed a bionic three-region GBM model that could be used to explore GBM disease mechanisms and screen drug compounds **(**Fig. 8C**)**. Rengaraj et al. [209] combined 3D printed micro scaffolds fabricated using two-photon polymerization technology with bioactive thin-film coatings to build organoid models. They used these models to systematically compare the behavior of two human pancreatic cell lines, PAN092 (patient-derived cell line) and PANC1 (immortalized cell line), thus revealing their responses to membrane stiffness and stroma-bound bone morphogenetic proteins. Their results demonstrated that this approach is suitable for forming microscale tumor tissues and modeling the early stages of metastatic cancer** (**Fig. 8D**)**.

Boot et al. [210] presented a large animal model of chronic methicillin-resistant *Staphylococcus aureus*. In this model, an injectable heat-sensitive HA-based hydrogel containing vancomycin and gentamicin was found to be superior to other treatments, eliminating bacteria from all animals. Maloney et al. [206] demonstrated an immersion printing technique using HA and collagen hydrogels as bioinks. This technique prevented the bioink from interacting with the well walls and provided a support to maintain the sphericity of the printed structure. The authors successfully used this technique to bioprint tissue-like organs in 96-well plates to improve throughput for 3D drug screening. Since it was first reported, this technique has facilitated oncology drug-screening studies and represented a significant advancement in clinical practice. A bio-orthogonal nanoengineering strategy [211] for the construction of photo-thermodynamic tumor spheroids has been developed, which not only provides a promising platform for studying tumorigenesis and therapeutic responses. The biosynthesis machine will be used to rapidly construct heterogeneous tumor spheroids through multivalent click ligand (ClickRod) reactions. ClickRod consists of HA-functionalized gold nanorods (AuNRs), which is a major component of the tumor extracellular matrix, and creates an optimal microenvironment to promote tumor growth and angiogenesis.

### Drug delivery

**Fig. 9:**
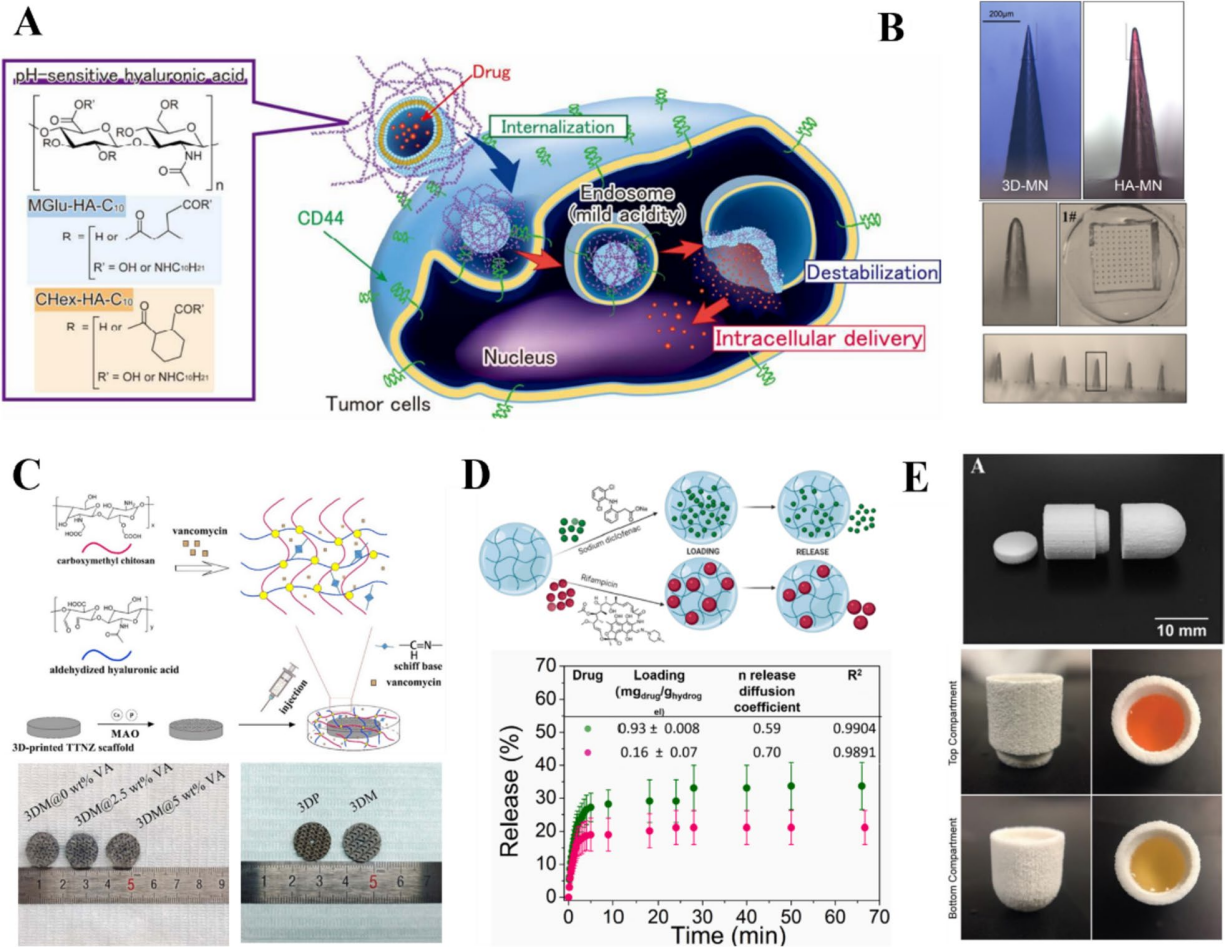
HA for drug delivery. **A** HA-derived modified liposomes are administered intracellularly to sites expressing CD44 cells. Reprinted (adapted) with permission from [215]. Copyright 2018, American Chemical Society. **B** 3D microneedles containing 6-r-hirudin [216]. Copyright 2022, the Authors. Published by IOP Publishing Ltd. Based on Creative Commons Attribution License (CC BY). **C** Vancomycin-containing HA-based hydrogel withTi-10Ta-2Nb-2Zr (TTNZ) stent and broad view of 3D-printed samples (3DP) and 3D printed scaffolds treated by MAO (3DM) stents [72]. Copyright 2021, Elsevier B.V. All rights reserved. **D** HA/chitosan hydrogels for 3D printing with modulation and facilitation of multiple drug release [217]. Copyright 2021, Elsevier B.V. All rights reserved. License Number: 5620831345362. **E** Preformed tablet chamber for the treatment of hypertension [218]. Copyright 2018, the Authors. Licensee MDPI, Basel, Switzerland. Based on Creative Commons Attribution License (CC BY)

HA nanoparticles are significantly advantageous for targeted drug delivery. HA acts as a delivery vehicle that protects the drug, prevents premature drug inactivation, and delays drug release, thereby ensuring that the drug reaches the target site at a fixed time and location to achieve targeted therapy. Notably, the solubility of the drug can be improved by direct binding to HA. Furthermore, HA can reduce plasma clearance, thus prolonging the half-life of the drug [212]. Based on the binding of HA to CD44 receptors, HA-based nanoparticles have been evaluated for the development of targeted cancer therapies that can selectively transfer drugs to cancer cells through enhanced permeability and retention. In this way, adverse effects can be controlled and targeted drug delivery can be achieved [213, 214]. For example, antitumor drugs, such as doxorubicin, are lipophilic and insoluble in water. Self-assembled HA-based nanoparticles have been shown to be effective for targeting CD44-positive cancer cells (Fig. 9A).

HA has good water solubility but is insoluble in most organic solvents, which limits its modification by esterification using oil-soluble hydroxyl compounds. The hydrophobic monomer paclitaxel was grafted onto HA by esterification using polyethylene glycol nanocomposite technology [215, 219]. Chitosan and HA were used to develop multiplexed hydrogels with tailored properties and controlled drug release. Meng et al. [220] designed a bilayer microneedle consisting of HAMA, HA, and polyvinylpyrrolidone (PVP). Compared to single-layer microneedles, the bilayer HAMA-HA-PVP microneedles acted as effective drug reservoirs for drug release in the presence of water. This eliminated the need for multiple injections, thereby improving patient compliance** (**Fig. 9B**)**. Huang et al. [72] developed a low elastic modulus Ti-10Ta-2Nb-2Zr (TTNZ) alloy based on vancomycin-loaded hydrogels combined with 3D-printed through-hole porous titanium alloy scaffolds to impart antimicrobial properties to 3D-printed TTNZ scaffolds. The results showed that the loading of 2.5 wt.% and 5 wt.% vancomycin had no effect on the structure of chitosan HA hydrogels. When combined with the porous scaffold, the drug-loaded hydrogels exhibited a slower drug release rate and longer release time than the normal hydrogels** (**Fig. 9C**)**. Based on the intermolecular interactions, Maiz-Fernandez et al. [217] optimized the adhesion, swelling, biodegradation, mechanical, and rheological properties of HA/chitosan polyelectrolytes by adjusting parameters, such as the polysaccharide content and coordination time** (**Fig. 9D**)**. In this way, the researchers could regulate and promote the controlled release of a wide range of drugs, such as the anionic and anti-inflammatory diclofenac sodium and the neutral antibiotic rifampicin.

HA gel microspheres are generally prepared under aqueous conditions via ionic crosslinking to encapsulate cells, growth factors, and bioactive proteins. Thus, gels and solid microspheres made from HA are readily available for use as delivery systems for drugs, growth factors, and cells. Wang et al. [71] explored the on-demand 3D printing of pills with personalized drug dosages. This technology also enabled the creation of pills with more complex dosages to improve patient compliance by reducing the number of pills required per dose. Acosta-Vélez et al. [218] designed a combined therapeutic oral dosage scheme for the treatment of hypertension** (**Fig. 9E**)**. The formulation used a photocurable hydrophilic HA-based bioink and photocurable hydrophobic polyethylene glycol bioink loaded with lisinopril and spironolactone. This study demonstrated the feasibility of combining therapeutic oral dosage forms, particularly for drugs for which the pharmacological effects can be achieved at low doses.

### Other applications

3D printable HA bioinks have also been used in other emerging tissue engineering applications. For example, 3D bioprinting of cardiovascular components is often conducted using decellularized ECM bioinks. HA can be added to these bioinks to maintain the homeostasis of the extracellular environment. Tuning the mechanical and rheological properties of HA-based hydrogels by simple modification or crosslinking methods has enabled researchers to use them for tissue engineering and bioprinting of various tissues, such as retina, uterus, and laryngeal cartilage. Specifically, bioprinted constructs have been shown to be biocompatible in animal models. In addition, HA-based hydrogels as injectable cell carriers to improve myocardial perfusion [221]. Zhang et al. [222] developed a 3D PCL scaffold containing an HA hydrogel layer that significantly shortened the length of the vascular-like network and contributed to the formation of lumen and actin networks. The hybridized crosslinked hydrogel (CHA/CHX), an HA-based antimicrobial microgel, exhibited good injectability and deformability. Moreover, it exhibited good biocompatibility and remarkably effective antimicrobial action, thus making it a promising material for the treatment of pacemaker implant infections [223]. Kreimendahl et al. [73] used fibrin and HA as a one-component hybrid bioink for freeform reversible embedding of suspended hydrogels (FRESH) bioprinting. The suspended hydrogel bioink with 1.0% fibrin and 0.5% HA provided optimized vascularization for low viscosity and low polymerization solution printing with good spatial resolution.

**Fig. 10:**
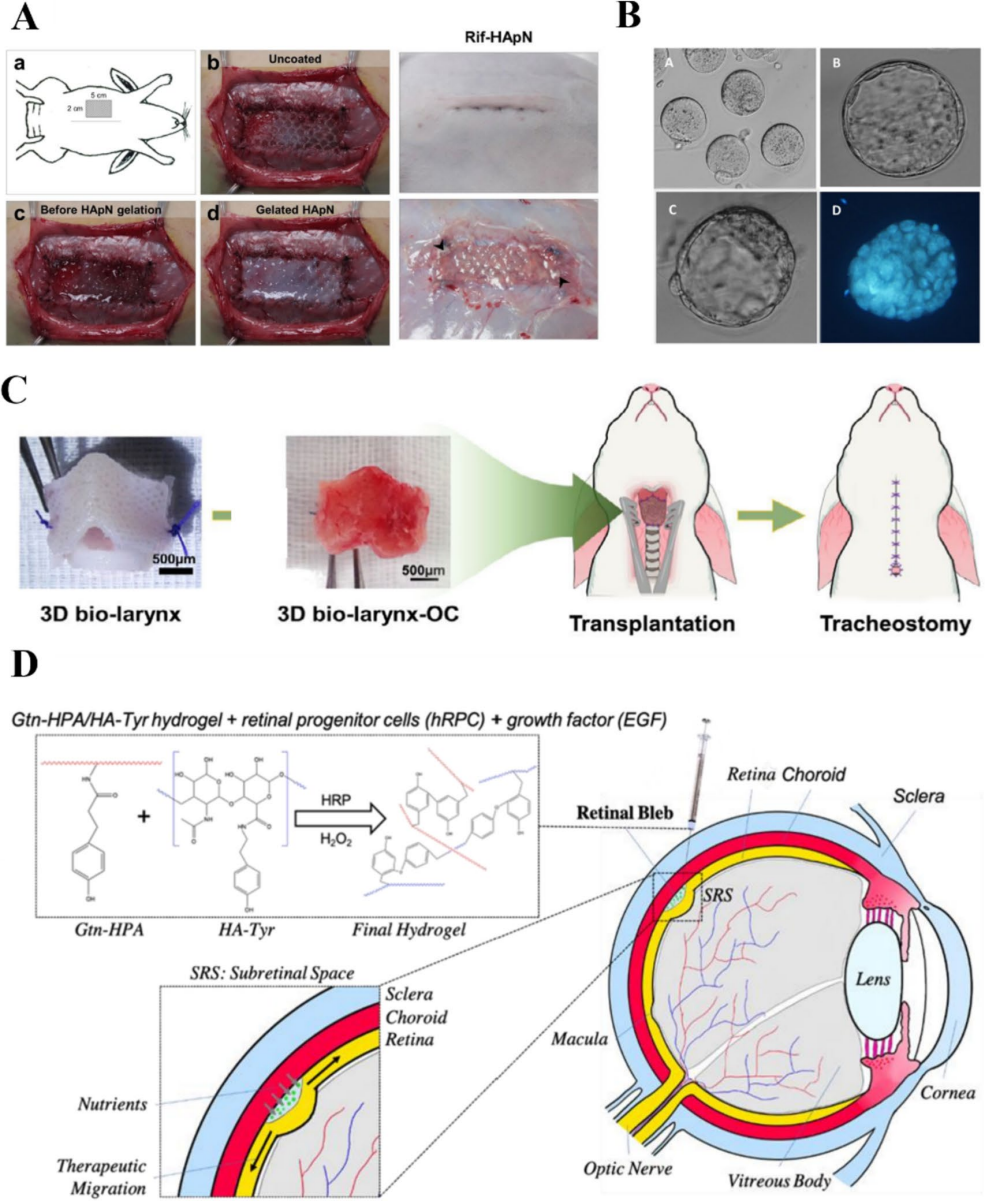
HA for other tissue engineering applications. **A** HA hydrogel as an anti-infective coating for in vivo patch implants [224]. Copyright 2020, the Authors. Licensee MDPI, Basel, Switzerland. Based on Creative Commons Attribution License (CC BY). **B** In vitro culture of HA-encapsulated oocytes [225]. Copyright 2022, the Authors. Based on Creative Commons Attribution License (CC BY). **C** HA for 3D bioprinting of artificial larynxes [226]. Copyright 2022, the Authors. Published by Wiley Periodicals, Inc. Based on Creative Commons Attribution License (CC BY). **D** Effect of HA-Tyr/GelMA hydrogels on human retinal progenitor cells (hRPCs) [228]. Copyright 2021, the Authors. Licensee MDPI, Basel, Switzerland. Based on Creative Commons Attribution License (CC BY)

Pérez-Köhler et al. [224] developed a thermally responsive rifampin-loaded HA hydrogel for implantation. This hydrogel had excellent antimicrobial effects in a rabbit hernia repair model. Moreover, it was degradable, thermally responsive, and antimicrobial, which facilitated its use in combination with prostheses in the surgical field** (**Fig. 10A**)**. Desai et al. [225] cultured 3D functionally competent metaphase II oocytes in vitro using a novel Tyr-linked HA. The resultant HA-based hydrogel acted as an efficient and versatile ECM-like biomaterial for the culture of 3D follicles. This culture model allowed for the ovulation of functionally normal mid-stage II oocytes capable of fertilization, genome activation, and blastocyst formation** (**Fig. 10B**)**. 3D bioprinting has become one of the most promising biomanufacturing technologies because of its advanced precision, intelligent flexibility, and tailored features. Impressive achievements have been made in various areas, and future applications are expected to become more widespread. Park et al. [226] used a (HAMA)/(GelMA) hybrid bioink with a porous polycaprolactone (PCL) outer framework to achieve structural strength of the printed structures and provide a suitable microenvironment to support the printed cells **(**Fig. 10C**)**. They established a new fluid supply system that can provide both the base medium and the 3D bioprinting process, thereby improving cell survival during printing. Animal experiments confirmed that the transplanted 3D biolarynx successfully maintained the airway, which has great potential in creating a biologically functional artificial larynx for laryngectomy patients. Nie et al. [227] used 3D extrusion-based bioprinting to construct a bilayer endometrial construct (EC) based on a sodium alginate-hyaluronic acid (Alg-HA) hydrogel for functional regeneration of the endometrium. The bilayer EC not only restored the morphology and structure of the endometrial wall (including organized lumen/glandular epithelium, stroma, vascular system, and smooth muscle layer) but also significantly improved reproductive outcomes in the post-implantation surgical region (75%, 12/16, *p* < 0.01).

3D bioprinting can also facilitate the treatment of ophthalmic diseases. Dehghani et al. [74] developed and applied 3D printed gel/elastin/HA membranes for conjunctival reconstruction. Dromel et al. [228] explored the effects of GelMA hydrogels on human retinal progenitor cells (hRPCs) and created an interpenetrating network polymer capable of encapsulating hRPCs. By regulating the stiffness of the hydrogel, the differentiation potential of hRPCs can be controlled. Interpenetrating network 75 (ipn75; 75% HA) resulted in higher expression of rod photoreceptor markers, while the expression of cone photoreceptor markers was higher in ipn50 **(**Fig. 10D**)**. Shrestha et al. [229] used HA-based biomaterials for the in vitro 3D modeling of retinal diseases and found that the addition of HA to gelatin scaffolds increased the cell viability and promoted the expression and characteristic morphology of neuronal phenotypes, including Tuj-1.

Karam et al. [230] have shown that soluble high molecular weight (HMW) HA increased the viability and tube formation of human brain microvascular ECs (HCMVECs). When HCMVECs were cultured on HA microporous annealed particulate scaffolds (HMAPS) with crosslinked HA of different molecular weights, the cellular response was comparable to that when soluble HA was cultured. The HMAPS of HMW HA were more vascularized than the HMAPS of LMW HA.

## Conclusions

This paper reviewed the applications of HA-based bioinks for 3D bioprinting. First, the production pathways, modification methods, and crosslinking methods for HA were described. Then, the advantages and disadvantages of different bioprinting methods for HA-based bioinks were discussed. Finally, the common applications of HA bioinks in the medical field were highlighted, thus demonstrating the potential of 3D printed HA biostructures.

HA is a natural hydrophilic polymer with several important properties, such as biocompatibility, anti-inflammatory properties, cytocompatibility, biodegradability, and mucosal adhesion. Several in vitro and in vivo studies have revealed the beneficial effects of HA/HA-based biomaterials and demonstrated their remarkable biological properties [231]. Although HA is widely used in tissue engineering, several issues remain to be resolved, including determining how to promote the adhesion, proliferation, and differentiation of osteoblasts for angiogenesis and bone tissue formation; how to mimic native structures and angiogenesis, especially in deeper regions of 3D structures (such as deep brain regions); how to deal with cell membrane-specific receptors, such as CD44 and RHAMM, interacting with HA to activate cellular signaling pathways and regulate cellular functions [232] and how to control the porosity of the hydrogels to ensure the transport of nutrients and metabolites, and activity and proliferation of cells [233].The exact mechanism of this binding and its downstream signaling mechanisms (e.g., receptor clustering, affinity) remain unclear that requires further investigation. In recent years, the poor mechanical properties of HA hydrogels have been compensated for to some extent by modifying natural polymers and combining them with synthetic polymers, inorganic materials, and scaffold materials (e.g., nanoclay, nanoparticles, fixed value implants, targeted drugs) [126, 234]. However, 3D bioprinting still faces many technical problems, such as scaffold degradability, short half-life of HA scaffold, poor gel kinetics, uncontrollable degradation rate, and poor cell viability. Technological enhancements in printing strategies will significantly benefit improving printing performance. 4D bioprinted structures capable of responding to internal cellular forces or external stimuli may provide better regenerative functionality of tissues and organs. Thus, there is still a long way to go before clinical applications of 4D bioprinting can be realized [235].

HA can be produced through tissue extraction, microbial fermentation, and artificial enzymatic synthesis methods. As the scope of HA applications has expanded, so has the consumer demand. This has led to a need for new processes to obtain HA with higher purity, productivity, and molecular weight [232]. However, efficient recombinant cell factories are being established, and in vitro cell-free production systems are being developed. several technical issues associated with the design and construction of super HA-producing strains must be resolved [96, 236].

A crucial advantage of the chemical modification of HA is that the resulting gel-based derivatives retain their shape and degrade more slowly than natural HA. The rate of enzymatic degradation can be controlled by varying the degree of modification to optimize the residence time at specific sites in vivo. The advantages of HA-based hydrogels have been further explored by elaborating on the diversity of existing chemical modification and processing technologies. The continued development of multifunctional biocompatible materials has become an important goal for researchers [98]. With the development of advanced crosslinking chemistry and further research on HA structural and functional modifications, newer biomanufacturing techniques will be developed that will promote the wider use of HA bio-linkages in regenerative medicine [66].

To the best of our knowledge, the sterilization of bioinks for 3D bioprinting has not been investigated. Sterilization of bioinks will inevitably cause changes in the material, such as material degradation, discoloration, embrittlement, and odor generation, and it may also promote further crosslinking or induce toxic effects that may impair the performance of the bioink[237]. Ethylene oxide (EtO) sterilization is a method of minimizing the degradation of HA hydrogels. Despite the importance of sterilization, its effect on hydrogel performance has not been fully studied [238]. In the future, the sterilization method of HA bio-ink should also be further studied to find a better way that has a less impact on the gel.

In summary, HA-based bioinks are promising 3D bioprinting materials that can be used for tissue repair and synthetic organ printing. Future developments in biomaterials science and innovative breakthroughs in 3D printing technologies will open a new chapter in the field of human tissue repair and regeneration.

## Data Availability

Data sharing not applicable to this article.

## Abbreviations

HA: Hyaluronic acid
ECM: Extracellular matrix
Bioinks: Biomaterial-based inks
MSCs: Mesenchymal stem cells
HAS: Hyaluronidase
EC: Endothelial cell
SA: Transpeptidase sortase A
HA-TYR: HA-tyramine
DLP: Digital light processing
DX: Doxycycline
PVA: Polyvinyl alcohol
HyA: Hydroxyapatite
HBC: Hydroxypropyl chitosan
HAVS: Vinyl sulfonated hyaluronic acid
HASH: Thiolated hyaluronic acid
Nscs: Neural stem cells
PLA: Poly-lactic acid
HA-PBA: Phenylboronic acid modified hyaluronic acid
hDPSCs: Dental pulp stem cells
HUVECs: Human umbilical vein endothelial cells
Gel: Gelatin
HAMA: Hyaluronic acid methacrylate
HACA: Catechol modified hyaluronic acid
GelMA: Gelatin methacryloyl
